# Participatory Methods to Engage Health Service Users in the Development of Electronic Health Resources: Systematic Review

**DOI:** 10.2196/11474

**Published:** 2019-02-22

**Authors:** Gaye Moore, Helen Wilding, Kathleen Gray, David Castle

**Affiliations:** 1 Mental Health Executive Services St Vincent's Hospital, Melbourne Fitzroy Australia; 2 Department of Nursing Faculty of Medicine, Dentistry and Health Sciences University of Melbourne Melbourne Australia; 3 Library Service St Vincent's Hospital Melbourne Fitzroy Australia; 4 Health and Biomedical Informatics Centre University of Melbourne Melbourne Australia; 5 Department of Psychiatry Faculty of Medicine, Dentistry and Health Sciences University of Melbourne Melbourne Australia

**Keywords:** eHealth, community-based participatory research, consumer participation, patient participation, program development, planning techniques, software design, internet, telemedicine, mobile apps

## Abstract

**Background:**

When health service providers (HSP) plan to develop electronic health (eHealth) resources for health service users (HSU), the latter’s involvement is essential. Typically, however, HSP, HSU, and technology developers engaged to produce the resources lack expertise in participatory design methodologies suited to the eHealth context. Furthermore, it can be difficult to identify an established method to use, or determine how to work stepwise through any particular process.

**Objective:**

We sought to summarize the evidence about participatory methods and frameworks used to engage HSU in the development of eHealth resources from the beginning of the design process.

**Methods:**

We searched for studies reporting participatory processes in initial development of eHealth resources from 2006 to 2016 in 9 bibliographic databases: MEDLINE, EMBASE, CINAHL, PsycINFO, Emcare, Cochrane Library, Web of Science, ACM Guide to Computing Literature, and IEEE Xplore. From 15,117 records initially screened on title and abstract for relevance to eHealth and early participatory design, 603 studies were assessed for eligibility on full text. The remaining 90 studies were rated by 2 reviewers using the Mixed Methods Appraisal Tool Version 2011 (Pluye et al; MMAT) and analyzed with respect to health area, purpose, technology type, and country of study. The 30 studies scoring 90% or higher on MMAT were included in a detailed qualitative synthesis.

**Results:**

Of the 90 MMAT-rated studies, the highest reported (1) health areas were cancer and mental disorders, (2) eHealth technologies were websites and mobile apps, (3) targeted populations were youth and women, and (4) countries of study were the United States, the United Kingdom, and the Netherlands. Of the top 30 studies the highest reported participatory frameworks were User-Centered Design, Participatory Action Research Framework, and the Center for eHealth Research and Disease Management (CeHRes) Roadmap, and the highest reported model underpinning development and engagement was Social Cognitive Theory. Of the 30 studies, 4 reported on all the 5 stages of the CeHRes Roadmap.

**Conclusions:**

The top 30 studies yielded 24 participatory frameworks. Many studies referred to using participatory design methods without reference to a framework. The application of a structured framework such as the CeHRes Roadmap and a model such as Social Cognitive Theory creates a foundation for a well-designed eHealth initiative that ensures clarity and enables replication across participatory design projects. The framework and model need to be clearly articulated and address issues that include resource availability, responsiveness to change, and the criteria for good practice. This review creates an information resource for future eHealth developers, to guide the design of their eHealth resource with a framework that can support further evaluation and development.

**Trial Registration:**

PROSPERO CRD42017053838; https://www.crd.york.ac.uk/prospero/display_record.php?RecordID=53838

## Introduction

### Rationale

Individuals are increasingly being offered access to health services via electronic health (eHealth), sometimes called digital health, that is, health-related electronic resources that connect them with health service providers (HSP) over the internet. Examples include websites, portals, social media sites, serious games, mobile apps, wearable self-monitoring devices, online learning sites, telehealth platforms, and shareable electronic health records. Patients, clients, or consumers in this review are called health service users (HSU). They may require services to support their physical health, mental health, and well-being in the broadest sense of the World Health Organization’s definition (Table 1) [1].

The involvement of HSU as full participants in eHealth innovations responds to a social movement that is over a decade old and influenced by many general trends in the digital economy and the information society [2]. Regardless of the form or purpose of eHealth resources, a common question is how HSP and HSU can optimally work together to design, build, and operationalize them; monitor their performance; and evaluate their impact [3].

Like most HSU, most HSP have little or no experience or training that equips them to collaborate effectively to develop eHealth resources, and so they are likely to turn to information technology professionals. However, technical developers or vendors who are commissioned to develop eHealth initiatives and technologies will turn back to their health sector partners for answers to who, what, where, when, why and how questions about engaging HSU in the early stages of the process. Furthermore, technical developers’ responsibilities usually end on delivery of a working product. Thereafter, HSP may or may not have clear ways of assigning responsibility for managing and governing the product’s use; in any case, HSU participation may be overlooked in these later stages in the life cycle of an eHealth resource. Participatory action research (PAR) [4] may be the launchpad for development [3], but at the end of development projects, there remains the need to operationalize and sustain the eHealth resources that have been created. The continuing quality assurance of eHealth resources within the auspicing health service also needs ongoing participation by HSU.

Apart from operational needs for HSU participation, there are ethical reasons for it. HSP have an ethical responsibility for ensuring that eHealth innovations achieve health outcomes for HSU. HSP are committed to evidence-based practice, in this as in other aspects of their work. Therefore, when they think about developing and deploying new eHealth resources, where do they find what is recognized as good practice in HSU participation? There are so many case studies that it is a near-impossible task to synthesize them all; furthermore, some *talk the talk* but do not *walk the walk* of HSU participation, some do not follow any recognized methodology, and some finish early in the life cycle of the eHealth resource.

There are numerous reviews and design guidelines that generalize about theories and methods of HSU participation in eHealth design. They emphasize the importance of the following basic principles:

- appreciation and understanding from the outset, of the range of potential HSU characteristics, goals, needs, values, and perspectives on use [5-7]
- attention to the needs of HSU not just as individual actors but also within their formal and informal care networks [8]
- careful alignment of diverse concerns, attitudes, and perspectives that expert content creators, HSP, and HSU may have [9-12]
- genuine active involvement so that HSU have an opportunity to identify practical problems and design, test, evaluate, and make decisions about technology in a range of environments [13-15]

At the same time, they note that methods of HSU participation in eHealth design need to use human and other project resources judiciously. The themes are as follows:

- ensuring that complex planning and evaluation models are able to be translated and streamlined to develop resources that are practical, feasible, and impactful in real-life settings [16]
- taking a systematic approach to requirements specification to avoid mismatch with the organizational context and to support summative evaluation on a feature-specific level [17]
- applying automation to expedite routine steps to create libraries of typical users and use cases and to manage unforeseen lessons learned for efficiency [18,19]

**Table 1:** Glossary of terms.

Term	Definition
Health	A state of complete physical, mental, and social well-being and not merely the absence of disease or infirmity, in accordance with the World Health Organization’s definition [1]
Health services	Organizations delivering support in the areas of physical, mental, or social well-being
Health service users	Patients, clients, or consumers who are recipients of health services. Excludes family or informal caregivers who receive health services solely in their caring role
Health service providers	Includes health professionals and health service managers
Electronic health (eHealth)	Health-related electronic resources, sometimes described as innovations, initiatives, applications, solutions, and tools
eHealth resources	Interactive electronic resources including websites, portals, mobile technology, mobile apps, blogs, social media, podcasts, wearable fitness or tracking devices, e-learning, telehealth, video, electronic health records, and software
Participatory methods	Systematic methods used to include end users as codevelopers. Includes participatory approaches, processes, and frameworks, such as participatory action research
Participatory processes	Specific steps taken to ensure engagement of end users, such as focus groups

Nevertheless, key considerations aside, it is difficult for HSP to identify from the literature a recognized, reliable methodological framework for engaging with HSU in the development of eHealth resources. A recent systematic review found that the literature variously encompassed 6 key phases and 17 different methods of participatory design, and it also found that sufficiency of reporting was poor and that no study undertook a robust assessment of efficacy [20]. This makes it difficult for HSP to study the effects of HSU participation in eHealth resources development on reach, adoption, acceptance, and efficacy of the intervention. Relative to other areas of health research, this type of study is immature, without widely endorsed methodological conventions for describing realistic aims for such projects or for determining valid measures of such effects [21].

Therefore, this paper investigates reports of eHealth applications and tools and resource development to determine what methods have been used systematically to ensure full HSU participation. We sought to distill evidence of positive, negative, or other unanticipated effects that have arisen at any stage in the eHealth resource life cycle from various HSU participation methods. Within these participatory approaches, we identified the reported impact from the point of view of HSU and HSP.

The impetus for this study began when the authors sought a strong research framework within which to undertake co-design of an eHealth initiative. The project was based on a print-based and workshop-based psychoeducational intervention called the Optimal Health Program (OHP). The authors wanted to ensure that they chose a rigorous methodological framework for redevelopment of OHP as an eHealth resource. Utilizing proven participatory methods would (1) optimize HSU engagement with the OHP resource that was developed, (2) strengthen the relevance of the resource to intended HSU, and (3) provide a logical foundation for long-term evaluation and improvement of the resource.

### Objectives

This paper reviews published research reports that include detailed descriptions of participatory methods to engage HSU in eHealth resource development projects. Through synthesizing answers to the following questions, the objective of this paper is to support critical evaluation of this type of methodology and informed selection of appropriate approaches in future research and development projects:

1. What types of eHealth resources have been developed using participatory processes, intended for what types of end users?
2. What frameworks have been used from the very beginning of the design process to ensure participation by the intended end users in the development of eHealth resources?
3. What methods within these frameworks have been most effective in supporting full involvement by intended end users of eHealth resources?
4. What aspects of the participatory methods in these eHealth projects have emerged as being most important to end users?
5. What positive, negative, or other unanticipated effects of participatory methods have the researchers reported at eHealth resource design, development, implementation, or evaluation stages?

## Methods

### Protocol and Registration

This systematic review has been carried out in accordance with the Preferred Reporting Items for Systematic Reviews and Meta-Analyses (PRISMA) statement [22,23]. Protocol CRD42017053838 was lodged with the PROSPERO international prospective register of systematic reviews.

### Information Sources

A total of 9 bibliographic databases were searched, including 6 health and biomedical databases and 3 technology databases:

- Ovid MEDLINE(R) Epub Ahead of Print, In-Process & Other Non-Indexed Citations, Ovid MEDLINE(R) Daily and Ovid MEDLINE(R) 1946 to Present (“MEDLINE”)
- EMBASE (Embase.com ) (“EMBASE”)
- CINAHL Plus with Full Text (EBSCOhost) (“CINAHL”)
- PsycINFO 1806 to January Week 4 2017 (Ovid) (“PsycINFO”)
- Ovid Emcare 1995 to 2016 week 49 (“Emcare”)
- Cochrane Library, including Cochrane Database of Systematic Reviews; Database of Abstracts of Reviews of Effect; Cochrane Central Register of Controlled Trials; Cochrane Methodology Register; Health Technology Assessment Database; NHS Economic Evaluation Database; About the Cochrane Collaboration (“Cochrane”)
- Web of Science Core Collection (“Web of Science”)
- ACM Guide to Computing Literature (“ACM”)
- IEEE Xplore Digital Library (“IEEE”)

Additional articles were identified from reference lists of key articles and *cited by* references in Google Scholar.

### Search

Search strategies were developed by an experienced medical research librarian (HW) in consultation with the OHP project leader (GM) and an expert eHealth researcher (KG).

In December 2015, scoping searches were developed and run in MEDLINE, EMBASE, CINAHL, PsycINFO, and Cochrane. In April 2016, brief confirmatory searches were run in Google Scholar to consider gaps in the initial strategy and additional search terms or databases that could be included. As a result, search strategies were refined and rerun in the initial health and biomedical databases as well as 3 additional technology databases: Web of Science, ACM, and IEEE. In February 2017, searches were updated to include results to the end of 2016. At this stage, an additional health database, Emcare, was also searched.

Within the health and biomedical databases (MEDLINE, EMBASE, CINAHL, PsycINFO, Emcare, and Cochrane) the search strategies combined the general concepts of user participation AND electronic resources AND program design. These search strategies were not limited to health-related conditions or resources because they yielded a small proportion of nonhealth-related results that could be removed manually. This enabled a very wide range of health conditions, HSU, organizations, and resources to be included in the results.

Within the broader technology databases that are not health specific (Web of Science, ACM, and IEEE), the search strategies were necessarily limited to health-related resources, combining the general concepts of user participation AND electronic resources AND (health OR well-being) AND program design.

A detailed search strategy was developed for MEDLINE using a combination of Medical Subject Headings (MeSH) and text words (Textbox 1). This was then adapted for the other databases, taking into account relevant subject headings and syntax. Search results were limited to publications dated from January 2006 to December 2016 and publications in English language. All database searches were updated in February 2017. Final search strategies for all databases are provided in Multimedia Appendix 1.

**Search strategy for Ovid MEDLINE.**

Ovid MEDLINE(R) Epub Ahead of Print, In-Process & Other Non-Indexed Citations, Ovid MEDLINE(R) Daily and Ovid MEDLINE(R) 1946 to Present

1. Community-Based Participatory Research/ or consumer participation/ or patient participation/
2. (codesign* or co-design* or coproduc* or co-produc* or cocreat* or co-creat* or participatory or e-collaboration or usability or focus group*).ti,ab.
3. ((user* or patient* or consumer* or family or families or carer* or caregiver* or participant* or client* or stakeholder* or peer*) adj2 (centre* or center* or centric or involv* or participat* or partner* or activat* or experience or advisor* or includ* or inclusion or engag* or collaborat* or consult* or empower* or input* or led or focus*)).ti,ab.
4. 1 or 2 or 3
5. internet/ or blogging/ or social media/ or audiovisual aids/ or multimedia/ or cell phones/ or text messaging/ or webcasts as topic/ or Telemedicine/ or videoconferencing/ or educational technology/ or audiovisual aids/ or motion pictures as topic/ or multimedia/ or exp optical storage devices/ or radio/ or exp tape recording/ or exp television/ or Mobile Applications/ or Software Design/
6. (internet or web* or online or www* or audiovisual* or audio-visual* or multimedia or multi-media or ehealth or e-health or mobile tech* or mobile phone* or mobile device* or mobile health or mhealth or m-health or cell phone or cellular phone or smartphone or app or apps or blog* or social media or social network* or facebook or podcast* or tracking device* or electronic health device* or fitbit or elearning or e-learning or wearable device* or smartwatch* or wearable electronics or telemedicine or tele-medicine or telehealth or tele-health or video* or electronic patient record* or electronic medical record* or electronic health record* or electronic record* or wiki* or portal* or behavioural intervention technolog* or health information technolog* or software or medical informatic* or health informatic* or digital health).ti.
7. 5 or 6
8. Program development/ or planning techniques/ or equipment Design/ or software design/
9. (develop* or creat* or plan* or build* or implement* or codesign* or co-design*).ti,ab. or design*.ti.
10. 8 or 9
11. 4 and 7 and 10
12. limit 11 to (english language and yr=“2006 -2016”)

### Study Selection

The search results were exported from all bibliographic databases to Endnote bibliographic management software. Duplicates were identified and manually removed within Endnote by HW. The records were initially screened within Endnote on title and abstract by HW, excluding results that were clearly irrelevant, that is, not electronic technology, not health or well-being related, not development processes, or not involving end users. All potentially eligible records were exported from Endnote to Covidence, an online platform for managing the systematic review process. Covidence was used to screen records on title and abstract by any 2 of GM, HW, KG, and 1 additional reviewer using predefined inclusion and exclusion criteria as shown in Textboxes 2 and 3. All types of study design were eligible for initial inclusion.

Full-text articles were obtained and uploaded to Covidence for all the available records that had been included based on title and abstract. When a number of articles reported on the same project, they were grouped into a single study to be reviewed together. The full text was reviewed independently by 2 reviewers, GM and HW, using additional inclusion and exclusion criteria (Textboxes 4 and 5).

**Inclusion criteria for screening on title and abstract.**

- English language
- Author identified
- Abstract available
- Intended end users are health service users (HSU)
- Intended HSU are involved by proxies: patient associations/advocates/family caregivers
- End users over 14 years of age
- End users are involved in the initial design/development stages

**Exclusion criteria for screening on title and abstract.**

- Language other than English
- Author not identified
- Abstract unavailable
- Intended end users are health service providers (HSP) only
- Intended end users are patient advocates, informal caregivers, or family caregivers in their own right
- End users under 14 years of age
- End users are involved only in the later stages of development
- Dissertation
- Duplicate record

**Additional inclusion criteria for full-text review.**

- Full text available
- Full conference papers
- Sufficient information on early design/development
- Inclusion of end users on their own behalf
- Development of a specific electronic health (eHealth) resource
- The eHealth resource is designed to support HSU interaction

**Additional exclusion criteria for full-text review.**

- Full text unavailable
- Conference abstract only
- Insufficient information on early design stage
- Patient associations/advocates/family caregivers as spokespersons for health service users (HSU)
- No specific electronic health (eHealth) resource actually developed
- Text or video content resources only, with no additional interactivity beyond content consumption
- Hardware only
- Research methodology inappropriate
- Project aim unclear
- Review paper only

### Data Collection Process

The included studies were found to use qualitative, quantitative, and mixed methods for HSU participation in eHealth resource development; therefore, the Mixed Methods Appraisal Tool (MMAT) Version 2011 [24] was selected to analyze the rigor of these studies. The MMAT’s 19-assessment criteria were added to the extraction stage of Covidence. Each of the included full-text studies was assessed for methodological quality and rated according to the relevant MMAT criteria.

The detail of MMAT ratings under each criterion was extracted and recorded in an Excel spreadsheet. MMAT scoring metrics were used to calculate a total score for each study in order to develop a hierarchy of evidence for the strength of different methodologies (Multimedia Appendix 2).

The 2 reviewers, GM and HW, worked independently using MMAT to assess the methodological quality of papers and minimize risk of bias in assessing the literature. MMAT ratings and reasoning were compared, and conflicts were resolved through discussions between them.

#### Risk of Bias in Individual Studies

MMAT scores are typically 100%, 75%, 50%, and 25%. They work on the principle that a mixed-methods study is only as strong as its weakest part. This means that mixed-methods studies that have more criteria to meet (4 qualitative plus 4 quantitative plus 3 mixed method, equaling 11 criteria) could potentially be marked down more easily than studies that are purely qualitative and have fewer criteria to score (4 qualitative criteria only). In order to address this potential bias among study types, a decision was made to include an additional score of 90% to rationalize the difference that occurred between 100% and 75% in mixed-methods studies (Multimedia Appendix 2).

After assessment, studies were grouped by MMAT score and sorted into alphabetical order according to the surname of the first author. Although study numbers were initially used by the reviewers for identification purposes, these have been removed so that there is no confusion about study number and ranking. All studies with the same MMAT score hold equal ranking.

#### Data Items

The 90 studies assessed according to MMAT are summarized descriptively in a table (Multimedia Appendix 3). First, the data items described in Table 2 were manually extracted from the full text by HW and recorded in Excel for analysis. These results were grouped, tallied, and exported into separate tables according to characteristics of the research scope, such as health area, technology, population, or country of study (Multimedia Appendices 4-7).

Additional descriptive data were extracted from the full text of a subset of included studies, namely 30 studies that scored 90% or higher on MMAT. Data were extracted by HW and GM from the full text of each study using the data items listed in Table 3. These details were grouped, sorted, tallied, and exported into tables that summarize the main methods used to engage HSU in participatory development of eHealth resources.

Methods, frameworks, and processes varied enormously among studies; therefore, a decision was made to allocate all reported methods to the 5 stages of a single framework in order to standardize comparison. The Center for eHealth Research and Disease Management (CeHRes) Roadmap [25] was chosen for this purpose because it was specific to eHealth, highly cited (approximately 400 times between 2011 and 2017), based on the review of many eHealth and development frameworks, process oriented (not just a list of methods but a focus on specific steps), and defined within 5 stages.

Models and theories, participatory frameworks and interventions were extracted from the top 30 studies, and HW subsequently searched for additional mentions of them across the full text of the 90 MMAT–rated studies within Endnote.

#### Risk of Bias Across Studies

To minimize journal bias, a wide range of bibliographic databases were searched, including those with either a health focus or a technology focus. The search results were limited to English language, which could have created a cultural bias in the studies, although the 90 studies included in the quantitative analysis took place across 21 countries.

**Table 2:** Data items extracted from 90 studies.

Variable	Definition
Health area	Main area of health or well-being that the technology addresses; for example, cardiovascular diseases, mental disorders. Defined using MeSH^a^ terms—a controlled vocabulary of hierarchical subject headings from MEDLINE
Purpose	Purpose of the technology; for example, motivation, self-care, or health education. Defined using MeSH Terms—a controlled vocabulary of hierarchical subject headings from MEDLINE
Technology type	Identified technology developed; for example mobile app or website. If more than one, all technologies were recorded
Age group	Age group targeted by the resource (not to be confused by the age group of participants in the development process). Simplified into 3 groups: youth (12-24 years), adult (25-64 years), and aged (65+ years). eHealth^b^ projects aimed at children under 14 years were excluded; therefore, this age group was not included
Gender specific	Gender specific target of a resource; for example, female only or male only. Not recorded if the resource was inclusive of all genders rather than gender specific
LGBTQI+^c^ specific	LGBTQI+ specific target of a resource; for example, men who have sex with men. Not recorded if resource was inclusive rather than LGBTQI+ specific
Cultural/multicultural	Research focusing on a particular culture or across a number of different cultures (for example, Indigenous Australians). Not recorded if culture was not reported as an issue; for example, Swedish research taking place in Sweden with Swedish-speaking participants would not be included unless it was also researched in another country with another language for crosscultural comparison
Country where studied	Country where the research took place. If more than one, all are included

^a^MeSH: Medical Subject Headings.

^b^eHealth: electronic health.

^c^LGBTQI+: Lesbian, Gay, Bisexual, Transgender, Queer or Questioning, and Intersex+.

**Table 3:** Additional data items extracted from the top 30 studies.

Variable	Definition
Specific product	Specific resource developed; for example, named mobile app or website URL
Models and theory base	Defined structures and models within the project design and delivery, such as Stages of Change
Participatory frameworks	Defined frameworks involving end users in the development of resources, such as, CeHRes^a^ Roadmap
Interventions	Specific therapeutic program or guideline, such as Acceptance and Commitment Therapy
Health service user (HSU) population	Defined end user group for a particular eHealth^b^ project; for example, young people with diabetes
Health service provider (HSP) population	Defined group of health professionals involved in an eHealth project; for example, mental health clinicians, oncologists
Teams or groups	Defined teams or groups involved in the development of an eHealth project; for example, leadership team, research group, or advisory group
Methods	Methods or processes used during the development of an eHealth resource. Includes both participatory and nonparticipatory methods (for example, ethics application and literature search). Participants are identified for some methods (for example, Focus Group [HSU] and Interview [HSP]_
CeHRes Roadmap stage	Methods sorted into different stages of a defined participatory framework known as the CeHRes Roadmap [25]. The 5 stages include: (1) Contextual Inquiry, (2) Value Specification, (3) Design, (4) Operationalization, and (5) Summative Evaluation.
Themes/findings (HSU’s perspective)	Reported feedback from HSU about the eHealth resource and development process
Author/researcher recommendations	Reported results, limitations, and recommendations

^a^CeHRes: Center for eHealth Research and Disease Management.

^b^eHealth: electronic health.

The development of eHealth resources is a long process, sometimes taking many years, and many publications only reported a portion of the process, with only a few reporting the entire project up to final evaluation. As conference abstracts and grey literature were excluded in favor of journal articles, sections of the development process may have been reported elsewhere but not included in our evaluation. Reference lists and *cited by* references in Google Scholar were searched with respect to the top 90 studies to locate connected publications reporting later stages of development, but it is possible that some publications were either missed or published after our review timeframe.

## Results

Database searches retrieved 24,674 records, which were exported to Endnote. Duplicates were removed by HW, leaving 15,117 records. These records were screened for broad relevance on title and abstract by HW and 13,096 records were excluded as clearly irrelevant. The remaining 2021 records were assessed for eligibility on title and abstract using the inclusion and exclusion criteria in Textboxes 2 and 3, and 1391 records were excluded.

**Figure 1:**
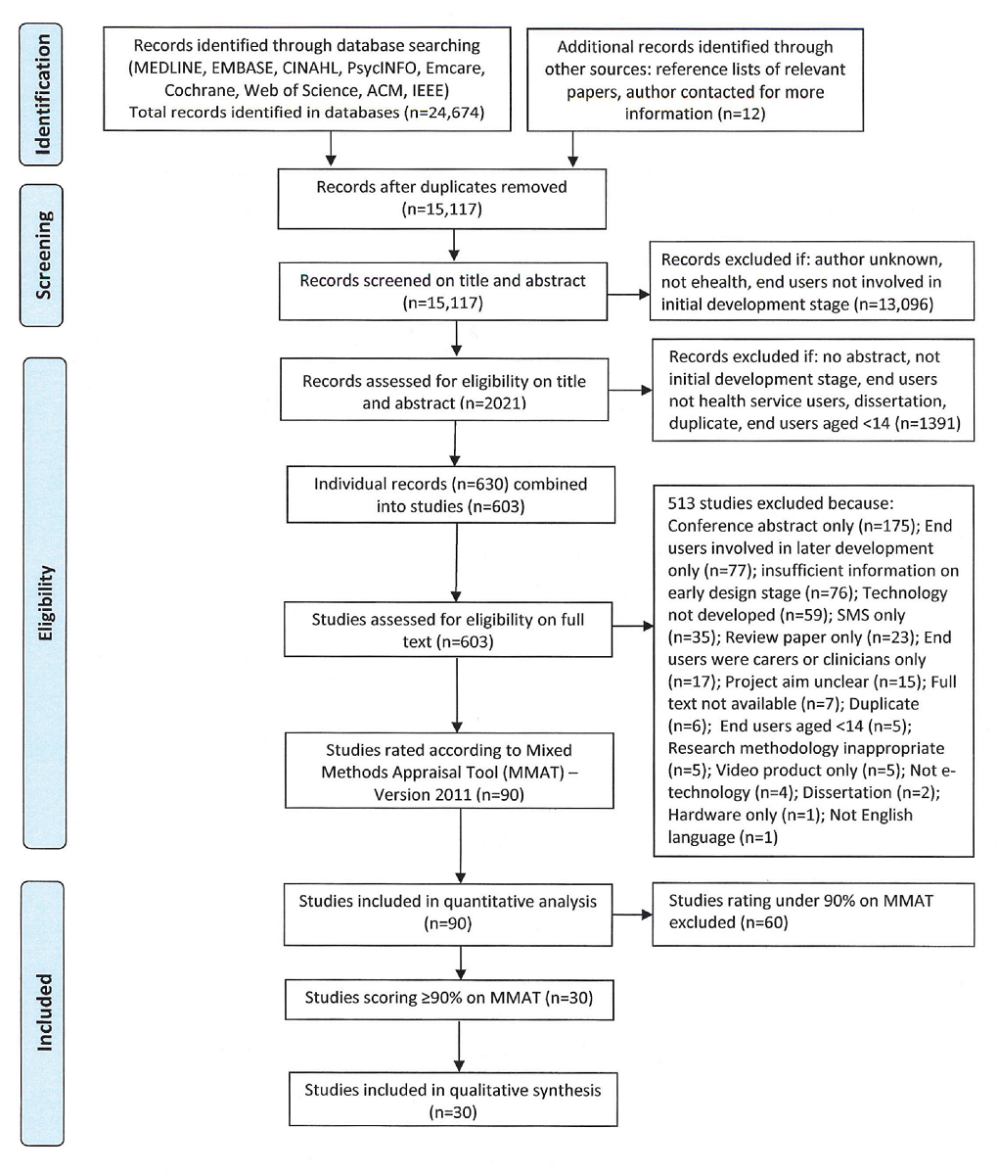
Preferred Reporting Items for Systematic Reviews and Meta-Analyses (PRISMA) flow diagram.

The 630 remaining records were combined into 603 studies, some of which involved multiple publications. All 603 studies were assessed for eligibility on full text, and 513 studies were excluded according to the criteria in Textboxes 4 and 5, leaving 90 studies for quantitative analysis. During the screening and full text review process, 12 additional records relating to the 90 studies were identified from reference lists or contact with authors, and those records were combined into the studies. See Figure 1 for the PRISMA flow diagram.

A total of 90 studies were assessed for quality according to MMAT. Results are summarized in Table 4 and detailed results are available in Multimedia Appendix 2. An MMAT score of 100% was awarded to 28 studies and 2 studies scored 90%.

### Results From 90 Studies Included in Quantitative Analysis

The 8 data items described in Table 2 were extracted from each of the 90 studies (Multimedia Appendix 3).

The major health focus of each study was grouped into a hierarchy of 18 wider MeSH subject headings, summarized in Multimedia Appendix 4. The top 5 health areas were neoplasms (cancer), mental disorders, nutritional and metabolic diseases (including weight management), virus diseases (including HIV), cardiovascular diseases, and endocrine system diseases (including diabetes).

Nine types of technology were reported in the 90 studies, and these are summarized in Multimedia Appendix 5. Websites (56 studies) and mobile apps (32 studies) were the main eHealth technologies developed. Other types of technology reported were decision tools, handheld computers, kiosk applications, personal health records, serious games, wearable devices, and telemonitoring.

Studies targeting specific populations are summarized in Multimedia Appendix 6. Of the 90 studies, 22 (24%) were youth specific, and 9 (10%) focused on the aged. Of the 90 studies, 11 (12%) reported eHealth projects for women only, and 4 (4%) were for men only. Moreover, 3 studies (3%) had a Lesbian, Gay, Bisexual, Transgender, Queer or Questioning, and Intersex+ focus. Fourteen studies (16%) had either a cultural or multicultural focus, such as a bilingual app for Indigenous Australians or the development of a website in both France and Finland.

The 90 studies took place in 21 countries, summarized in Multimedia Appendix 7. The top 6 countries were United States (33 studies), United Kingdom (15 studies), Netherlands (13 studies), Canada (7 studies), Sweden (6 studies), and Australia (6 studies). Studies also took place in Austria, Belgium, Czech Republic, Greece, Denmark, Finland, France, India, Spain, Ireland, Italy, New Zealand, Norway, Republic of Korea, and Saudi Arabia.

### Results From 30 Studies Included in Qualitative Synthesis

The 30 studies scoring 90% or higher on MMAT were recorded in Excel spreadsheets and reviewed in detail. Data items listed in Table 3 were extracted for each study (Multimedia Appendix 8).

The 30 studies are listed in Table 5, along with an indication of the CeHRes Roadmap stages reported. There was often a perceived overlap between stages 1 (contextual inquiry) and 2 (value specification) such as when focus groups may have covered both stages at once. Where this appeared to happen, it was reported in the spreadsheet and included in both stages in Table 5. Where the CeHRes Roadmap was particularly useful was in highlighting stages that were often not reported, such as operationalization or summative evaluation (Table 5). It is possible that some of these studies did address each stage but did not report them in journal articles that were reviewed.

A summary of the 30 highest MMAT–rated studies is represented in Table 6 with details of the product developed, technology used and targeted population. The health area and general purpose of each eHealth project, categorized using Medical Subject Headings (MeSH) is summarized in Multimedia Appendix 9.

The methods were recorded in Excel spreadsheets using the original terminology reported in each study. The details included the number of HSU or HSP involved in each process, the order of each activity as reported, and subprocesses within each method (for example, the type of design activity or workshop activity). These details are included in Multimedia Appendix 8. These detailed methods were then grouped so that they could be summarized using a consistent terminology and then compared. This summary of methods is included for each study in Table 7.

Models and theories referred to in the top 30 studies are shown in Table 8.

Tables 9-13 give an overview of the options used to satisfy each stage of the CeHRes roadmap and the popularity of these methods. Many of the methods reported may demonstrate formative evaluation processes occurring as part of an iterative process. We recommend referring to Multimedia Appendix 8 and the original references for additional information that may be able to identify the practical steps that were implemented.

**Table 4:** Summary of scoring of 90 studies according to the Mixed Methods Appraisal Tool Version 2011.

MMAT^a^ score (%)	Studies (n=90), n (%)	Articles (n=117), n (%)	Qualitative studies only (n=33), n (%)	Mixed-methods studies (n=57), n (%)	References
100	28 (31)	41 (35)	16 (48)	12 (21)	[26-66]
90	2 (2)	3 (3)	0 (0)	2 (4)	[67-69]
75	41 (46)	53 (45)	11 (33)	30 (53)	[70-123]
50	11 (12)	11 (9)	3 (9)	8 (14)	[124-134]
25	8 (9)	9 (77)	3 (9)	5 (9)	[5,135-143]

^a^MMAT: Mixed Methods Appraisal Tool Version 2011.

Lastly, the models and theories, participatory frameworks and interventions identified in the top 30 studies were searched across the full text of the 90 MMAT–rated studies within Endnote, and the results were ranked in order of prevalence in Multimedia Appendices 10-12.

Twenty three models or theories were identified from the 30 studies scoring 90% or higher on MMAT as playing a role in the development of eHealth resources. The most often reported models and theories were Social Cognitive Theory (n=4, 13%) [144], Theory of Planned Behavior (n=3, 10%) [145], Transtheoretical Model (Prochaska Stages of Change) (n=3, 10%) [146], and the Persuasive Technology Theory/Behavior Model for Persuasive Design (n=3, 10%) [147]. A large variety of other models and theories were referred to, with little overlap between studies. Full results are recorded in Multimedia Appendix 10.

A total of 24 named participatory frameworks or approaches were identified from the 30 studies scoring 90% or higher on MMAT (Multimedia Appendix 11). Only 20 of the 30 top scoring studies referred to a specific framework, with many studies referring more broadly to using participatory design or iterative design methods without reference to a particular named framework. The most often reported participatory frameworks or approaches were User-Centered Design ([UCD], n=5, 17%) [148], PAR framework (n=4, 13%) [149], CeHRes Roadmap (n=3, 10%) [25], Medical Research Council (MRC) Guide to Developing and Evaluating Complex Interventions (n=2, 7%) [150-152], and International Patient Decision Aid Standards Collaboration (n=2, 7%) [153].

Some studies referred to specific interventions that were integral to the function of the eHealth resource that was developed. Key interventions identified in Multimedia Appendix 12 were Cognitive Behavior Therapy (CBT), Behavior Change Techniques, and Mindfulness.

**Table 5:** Stages of the CeHRes Roadmap addressed in top 30 studies.

Study name and references	MMAT^a^ score (%)	Contextual inquiry	Value specification	Design	Operationalization	Summative evaluation
Ahtinen, 2013 [26]	100	✓	✓	✓	—^b^	—
Antypas,2014 [27]	100	✓	✓	✓	—	✓
Bengtsson, 2014 [28,29]	100	✓	✓	✓	—	—
Buccieri, 2015 [30]	100	✓	✓	✓	✓	—
Clayman, 2008 [31]	100	✓	✓	✓	—	—
Cordova, 2015 [32]	100	✓	✓	✓	—	—
Dabbs, 2009 [33]	100	✓	✓	✓	—	✓
Das, 2013 [34]	100	✓	✓	✓	✓	—
Davies, 2015 [35,36]	100	✓	✓	✓	✓	✓
Fennell, 2016 [39,44]	100	✓	✓	✓	✓	✓
Fonda, 2010 [40,41]	100	✓	✓	✓	—	—
Goldenberg, 2015 [42,43]	100	✓	✓	✓	—	—
Heckman, 2015 [45]	100	✓	✓	✓	—	✓
Kelders, 2013 [46]	100	✓	✓	✓	—	—
Lubberding, 2016 [37,38,47]	100	✓	✓	✓	✓	—
Meyer, 2007 [48]	100	✓	✓	✓	✓	✓
Miller, 2015 [49]	100	✓	✓	✓	—	✓
Morrison, 2015 [50]	100	✓	✓	✓	—	✓
O'Brien, 2016 [51]	100	✓	✓	✓	—	—
Peute, 2015 [52]	100	✓	✓	✓	—	—
Revenas, 2015 [53-55]	100	✓	✓	✓	—	—
Sandlund, 2015 [56]	100	✓	✓	✓	—	—
Schnall, 2016 [57]	100	✓	✓	✓	—	—
Skjoth, 2015 [58]	100	✓	✓	✓	✓	—
Stinson, 2014 [59]	100	✓	✓	✓	—	✓
van Bruinessen, 2014 [60,61]	100	✓	✓	✓	✓	✓
Widman, 2016 [62]	100	✓	✓	✓	—	✓
Winterling, 2016 [63-66]	100	✓	✓	✓	—	✓
Ennis, 2014 [67,69]	90	✓	✓	✓	✓	—
Fleisher, 2014 [68]	90	✓	✓	✓	—	✓

^a^MMAT: Mixed Methods Appraisal Tool Version 2011.

^b^No information.

**Table 6:** Product, technology, and population in top 30 studies.

Study and references	Product	Technology	Population
Ahtinen 2013 [26]	Living application, a wellness app to support physical activity	Mobile app	Adult, Finland and India
Antypas 2014 [27]	Skibotn Rehabilitation Center resource with personal profile, activity calendar, and SMS^b^ reminders	Website and mobile app	Adult, aged, Norway
Bengtsson 2014 [28,29]	Mobile phone self-report system for self-management of hypertension	Mobile app	Adult, aged, Sweden
Buccieri 2015 [30]	Supporting and Assisting Youth (SAY) mobile app for homeless youth	Mobile app	Youth, Canada
Clayman 2008 [31]	Cancercarelinks.org—Cancer Care Links for women with breast cancer	Website	Adult, aged, female only, USA^c^
Cordova 2015 [32]	Mobile app version of Storytelling for Empowerment (S4E)—HIV/sexually transmitted infections and drug abuse preventive intervention for primary care	Mobile app	Youth, USA
Dabbs 2009 [33]	Pocket Personal Assistant for Tracking Health (Pocket PATH) for lung transplant patients	Handheld computer	Youth, adult, aged, USA
Das 2013 [34]	electronic health portal for weight loss patients undergoing treatment	Website	Youth, adult, Norway
Davies 2015 [35,36]	*Hep B Story* —culturally appropriate bilingual mobile app for Indigenous Australians with hepatitis B	Mobile app	Adult, indigenous Australians, Australia
Fennell 2016 [39,44]	Country Cancer Support website	Website	Youth, adult, aged, Australia
Fonda 2010 [40,41]	My Diabetes Data Tracker gadget—prototype personal health app for diabetes self-management	Mobile app, personal health record	Adult, aged, USA
Goldenberg 2015 [42,43]	HIV prevention app for men who have sex with men (MSM^d^)	Mobile app	Youth, adult, male only, MSM, USA
Heckman 2015 [45]	Online skin cancer risk reduction intervention for young adults—UV4.me	Website	Youth, USA
Kelders 2013 [46]	Web-based intervention for prevention of depression, based on self-help book *Living to the full*	Website	Adult, Netherlands
Lubberding 2016 [37,38,47]	OncoKompas—online self-management application for cancer survivors Oncokompas.nl	Website	Adult, aged, Netherlands
Meyer 2007 [48]	studentdepression.org—student focused website for depression self help	Website	Youth, UK^e^
Miller 2015 [49]	Prostate Cancer Online Guide and Resource for Electronic Survivorship (PROGRESS)—Web-based education program for prostate cancer survivors finishing active treatment	Website	Adult, aged, male only, USA
Morrison 2015 [50]	*Living well with Asthma*-online resource for self- management of asthma	Website	Youth, adult, UK
O’Brien 2016 [51]	LEAP (Living, Eating, Activity, and Planning in retirement)—Web-based lifestyle intervention in retirement	Website	Adult, aged, UK
Peute 2015 [52]	Website for childhood cancer survivors	Website	Youth, Netherlands
Revenas 2015 [53-55]	*tRAppen* —Swedish app for self-management of physical activity in rheumatoid arthritis	Mobile app	Youth, adult, aged, Sweden
Sandlund 2015 [56]	Mobile exercise app to prevent falls in senior citizens	Mobile app	Aged, Sweden
Schnall 2014 [57]	HIV prevention for high risk men who have sex with men (MSM)	Mobile app	Youth, adult, male only, MSM, USA
Skjoth 2015 [58]	Web-based decision aid support for pregnant women to make informed choices about Downs Syndrome screening-graviditetsportalen.dk	Website, Decision Tool	Youth, adult, female only, Denmark
Stinson 2014 [59]	iCanCope with Pain—mobile based self- management program for youth with chronic pain	Mobile app	Youth, Canada
Van Bruinessen 2014 [60,61]	PatientTIME; 3 products: self-directed online communication tool, corresponding evaluation plan, and implementation plan. Empowering patients to communicate with HSP	Website	Youth, adult, Netherlands
Widman 2016 [62]	ProjectHeartforGirls.com—interactive Web program to improve sexual communication and reduce HIV/sexually transmitted disease risk in adolescent girls	Website	Youth, female only, USA
Winterling 2016 [63-66]	Fex-Can, fertility and sexuality following cancer	Website	Youth, Sweden
Ennis 2014 [67,69]	myhealthlocker - electronic personal health record for people with severe mental illness	Personal Health Record	Youth, adult, UK
Fleisher 2014 [68]	Web-based decision-making intervention in cancer clinical trials PRE-ACT (Preparatory Education About Clinical Trials)	Website	Adult, aged, USA

^a^MeSH: Medical Subject Headings.

^b^SMS: short message service text messaging.

^c^USA: United States of America.

^d^MSM: men who have sex with men.

^e^UK: United Kingdom.

**Table 7:** Participatory frameworks and summary of methods in top 30 studies.

Study	Participatory framework	Summary of methods
Ahtinen 2013 [26]	Constructive design research, persuasive system design model	Interview (HSU^a^), thematic analysis, observation, think aloud, design activity, focus group (HSU), focus group (HSP^b^), co-design workshop (HSU + HSP), affinity wall, magical gadgets, content creation (HSU), iterative development, prototype, prototype evaluation
Antypas 2014 [27]	—^c^	Focus group (HSU), thematic analysis, prototype, randomized controlled trial (RCT^d^)
Bengtsson 2014 [28,29]	—	Ethics approval, HSP/expert identification of need, Focus group (HSU), focus group (HSP), thematic analysis, literature search, initial mock-up, interview (HSP), iterative development process
Buccieri 2015 [30]	Spiral technology action research model, youth centered participatory action study	Advisory group (HSU), literature search, focus group (HSU), design workshop (HSU), design activity-sketching idea, content creation (HSU), prototype, prototype evaluation, promotional campaign
Clayman 2008 [31]	—	HSP/expert identification of needs, interview (HSU)—longitudinal series, thematic analysis, initial mock-up, iterative development process, prototype, prototype evaluation, questionnaire (HSU)
Cordova 2015 [32]	Agile software development, community-based participatory research, ecodevelopmental framework	Advisory group, focus group (HSU), interview (HSU), thematic analysis, initial mock-up, iterative development process, prototype
Dabbs 2009 [33]	User Centered Design	Literature search, survey (HSU), interview (HSU), observation, design workshop (HSU), iterative development process, prototype, prototype evaluation, screen capture, usability testing, testing final version, usability questionnaire, RCT
Das 2013 [34]	Human centered design, iterative participatory design, collaborative analysis of requirements and design	Ethics approval, HSP/expert identification of needs, interview (HSP), observation, thematic analysis, design workshop (HSU), design workshop (HSP), workshop evaluation, design activity—sketching ideas, interview (HSU), co-design workshop (HSU + HSP), iterative development process, prototype, prototype evaluation, think aloud, screen capture, usability testing, questionnaire (HSU), usability questionnaire, implementation at location used for case study
Davies 2015 [35,36]	Participatory action research framework	Ethics approval, interview (HSU), thematic analysis, focus group (HSU), initial mock-up (storyboard), iterative development process, prototype, translation back and forth, launch event, evaluation questionnaire
Fennell 2016 [39,44]	Participatory action research framework	Ethics approval, literature search, review other resources, survey (HSU), interview (HSU), thematic analysis, advisory group (HSU), iterative development process, prototype, prototype evaluation, questionnaire (HSU), promotional campaign, launch event, usage statistics collected (google analytics), feedback form /Web survey /follow up survey
Fonda 2010 [40,41]	User Centered Design	Focus group (HSU), thematic analysis, focus group (HSP), iterative development process, prototype, prototype evaluation, prototype demonstration
Goldenberg 2015 [42,43]	—	Ethics approval, focus group (HSU), focus group (HSP), interview (HSP), thematic analysis, prototype, prototype evaluation
Heckman 2015 [45]	—	Survey (HSU), interview (HSU), think aloud, focus group (HSU), focus group (HSP), iterative development process, content development (HSP), prototype, prototype evaluation, readability/health literacy evaluation by experts, cognitive interviewing (HSU), acceptability testing, usability testing, questionnaire (HSU), thematic analysis, pilot testing, RCT
Kelders 2013 [46]	CeHRes Roadmap, Human Centered Design	Literature search, focus group (HSP), interview (HSU), thematic analysis, rapid prototyping, initial mock up, iterative development process, prototype, think aloud, usability testing, cognitive walkthrough
Lubberding 2016 [37,38,47]	—	Ethics approval, interviews (HSU), interviews (HSP), thematic analysis, content creation (HSU), iterative development process, prototype, prototype demonstration, think aloud, usability testing, cognitive walkthrough, implementation plan, feasibility study
Meyer 2007 [48]	Action Research Framework, Action Research Spiral	Ethics approval, advisory group, interview (HSU), survey (HSU), focus group (HSP), content creation (HSU), initial mock up, prototype, questionnaire (HSU), launch event, usage statistics collected, feedback form/Web survey/follow up survey
Miller 2015 [49]	Iterative Design	Literature search, review other resources, HSP/expert identification of needs, focus group (HSU), interview (HSU), initial mock up, content creation (HSU), iterative development process, prototype, observation, think aloud, readability/health literacy evaluation by experts, usability testing, thematic analysis, RCT
Morrison 2015 [50]	User Centered Design, Medical Research Council Guide to Developing and Evaluating Complex Interventions	Ethics approval, literature search, HSP/expert identification of needs, focus group (HSU), focus group (HSP), thematic analysis, initial mock-up, interview (HSU), iterative development process, prototype, think aloud, RCT
O’Brien 2016 [51]	Iterative Design	Ethics approval, literature search, HSP/expert identification of needs, co-design workshop (HSU + HSP), design activity, thematic analysis, content creation (HSU), iterative development process, prototype evaluation, cognitive walkthrough
Peute 2015 [52]	User Centered Design, Iterative Development Methodology, Website Development Model for the Healthcare Consumer	Literature search, survey (HSU), thematic analysis, co-design workshop (HSU + HSP), initial mockup, iterative development process, prototype, think aloud, screen capture, usability questionnaire, heuristic evaluation (experts)
Revenas 2015 [53-55]	Participatory Action Research Framework, Experience Based Design, User Centered Design	Ethics approval, survey (HSU), focus group (HSU), thematic analysis, advisory group (HSU), focus group (HSP), co-design workshop (HSU + HSP), iterative development process, prototype, observation
Sandlund 2015 [56]	Form-IT, Participatory and Appreciative Action and Reflection, Soft Systems Thinking	Ethics approval, focus group (HSU), interview (HSU), design workshop (HSU), design activity, iterative development process, prototype, prototype evaluation, observation, questionnaire (HSU), thematic analysis
Schnall 2014 [57]	Information Systems Research Framework, Iterative Design, User Centered Design	Literature search, focus group (HSU), thematic analysis, design workshop (HSU), design workshop (HSP), design activity—sketching ideas, iterative development process, prototype, usability testing, questionnaire (HSU), usability questionnaire, heuristic evaluation (experts)
Skjoth 2015 [58]	CeHRes Roadmap, International Patient Decision Aid Standards Collaboration	Literature search, focus group (HSU), focus group (HSP), interview (HSU), interview (HSP), observation, thematic analysis, prototype, prototype evaluation, design reflects clinical pathway
Stinson 2014 [59]	User Centered Design, Iterative design	Ethics approval, survey (HSU), survey (HSP), thematic analysis, focus group (HSU), focus group (HSP), interview (HSU), prototype, RCT
Van Bruinessen 2014 [60,61]	Intervention Mapping Framework, User Centered Design, Context Mapping Framework, Stanford Guidelines for Web Credibility	Ethics approval, advisory group (HSU), survey (HSU), focus group (HSU), design activity, thematic analysis, iterative development process, think aloud, heuristic evaluation (experts), implementation plan, RCT
Widman 2016 [62]	—	Interview (HSU), thematic analysis, literature search, review of other resources, advisory group (HSU), focus group (HSU), content creation (HSU), iterative development process, prototype, think aloud, usability testing, RCT
Winterling 2016 [63-66]	CeHRes Roadmap	Ethics approval, advisory group, focus group (HSU), interview (HSU), thematic analysis, iterative development process, prototype, RCT
Ennis 2014 [67,69]	—	Ethics approval, advisory group (HSU), survey (HSU), focus group (HSU), interview (HSU), thematic analysis, prototype, prototype evaluation, feasibility study, HSU as cofacilitators
Fleisher 2014 [68]	International Patient Decision Aid Standards Collaboration	Survey (HSU), focus group (HSU), interview (HSU), design activity, content creation (HSU), prototype evaluation, observation, RCT, feedback form/Web survey/follow-up survey

^a^HSU: health service users.

^b^HSP: health service providers.

^c^No information.

^d^RCT: randomized controlled trial.

**Table 8:** Model or theory base in top 30 studies.

Study and references	Model/theory base
Ahtinen 2013 [26]	Prochaska Stages of Change, Social Cognitive Theory
Antypas 2014 [27]	Prochaska Stages of Change, Health Behavior Theory, Social Cognitive Theory, Health Action Process Approach, Regulatory Focus Theory
Bengtsson 2014 [28,29]	Common Sense Model
Buccieri 2015 [30]	Critical Social Theory of Youth Empowerment
Clayman 2008 [31]	—^a^
Cordova 2015 [32]	Empowerment Theory
Dabbs 2009 [33]	—
Das 2013 [34]	—
Davies 2015 [35,36]	Paasche-Orlow & Wolf's Model (causal pathways)
Fennell 2016 [39,44]	Prochaska Stages of Change, Health Belief Model, Social Cognitive Theory, Reasoned Action Model, Theory of Planned Behavior, Information-Motivation Behavioral Skills Model
Fonda 2010 [40,41]	—
Goldenberg 2015 [42,43]	—
Heckman 2015 [45]	Integrative Model of Behavior Prediction
Kelders 2013 [46]	Persuasive Technology Theory, Business modeling
Lubberding 2016 [37,38,47]	—
Meyer 2007 [48]	Social Constructionist Philosophy
Miller 2015 [49]	Behavioral Science Theory, Cognitive-Social Health Information Processing Model
Morrison 2015 [50]	Normalization Process Theory
O’Brien 2016 [51]	Health Action Process Approach
Peute 2015 [52]	—
Revenas 2015 [53-55]	—
Sandlund 2015 [56]	Appreciative Inquiry
Schnall 2014 [57]	—
Skjoth 2015 [58]	--
Stinson 2014 [59]	Social Learning Theory, Behavioral Activation
Van Bruinessen 2014 [60,61]	—
Widman 2016 [62]	Fuzzy-trace Theory, Reasoned Action Model
Winterling 2016 [63-66]	—
Ennis 2014 [67,69]	—
Fleisher 2014 [68]	Cognitive-Social Health Information Processing Model, Ottawa Decision Support Framework

^a^No model or theory base described.

**Table 9:** Methods used in stage 1 (contextual enquiry) of the CeHRes Roadmap—top 30 studies.

Process/method	Total studies (n=30), n (%)	References
Ethics approval	15 (50)	[28,29,34-39,42-44,47,48,50,51,53-56,59-61,63-67,69]
Advisory group involved (HSU^a^)	6 (20)	[30,32,48,60,61,63-67,69]
Literature search	10 (30)	[30,33,39,44,46,49-52,57,58]
Review other resources	2 (7)	[39,44,49]
HSP^b^ or expert identified needs	6 (20)	[28,29,31,34,49-51]
Survey (HSU)	9 (30)	[33,39,44,45,52-55,59-61,67-69]
Survey (HSP)	1 (3)	[59]
Focus group (HSU)	13 (43)	[27-30,32,40-43,50,53-58,63-67,69]
Focus group (HSP)	5 (17)	[28,29,42,43,46,50,58]
Interview (HSU)	13 (43)	[26,31-33,35-39,44,45,47,48,56,62-67,69]
Interview (HSP)	3 (10)	[34,37,38,42,43,47]
Observation	2 (7)	[33,34]
Think aloud	1 (3)	[45]
Thematic or data analysis	18 (60)	[26-29,31,32,34-44,47,50,52,57,59,62-67,69]

^a^HSU: health service users.

^b^HSP: health service providers.

**Table 10:** Methods used in stage 2 (value specification) of the CeHRes Roadmap—top 30 studies.

Process/method	Total studies (n=30), n (%)	References
Advisory group involved (HSU^a^)	6 (20)	[30,32,48,60,61,63-67,69]
Literature search	2 (7)	[28,29,62]
Review other resources	1 (3)	[62]
Survey (HSU)	1 (3)	[48]
Focus group (HSU)	17 (57)	[27-30,32,35,36,40-43,45,49,50,53-56,58-61,63-66,68]
Focus group (HSP^b^)	5 (17)	[28,29,42,43,48,50,59]
Interview (HSU)	10 (33)	[26,31,32,37-39,44,46,47,49,58,62,67,69]
Interview (HSP)	1 (3)	[58]
Observation	3 (10)	[26,33,58]
Design workshop (HSU)	3 (10)	[34,56,57]
Design workshop (HSP)	1 (3)	[34]
Co-design workshop (HSU+HSP)	2 (7)	[51,52]
Workshop evaluation	1 (3)	[34]
Think aloud	1 (3)	[26]
Design activity	5 (17)	[26,34,51,56,60,61]
Thematic or data analysis	17 (57)	[26,27,34,37-44,46,47,50,51,53-55,57-62,67,69]
Content creation (HSU)	1 (3)	[48]
Rapid prototyping	1 (3)	[46]
Initial draft or simple mock up	9 (30)	[28,29,31,32,35,36,46,48-50,52]

^a^HSU: health service users.

^b^HSP: health service providers.

**Table 11:** Methods used in Stage 3 (design) of the CeHRes Roadmap—top 30 studies.

Process/method	Total studies (n=30), n (%)	References
Advisory group involved (HSU^a^)	7 (23)	[30,32,39,44,53-55,60-66]
Focus group or group discussion (HSU)	9 (30)	[26,30,40-43,45,53-55,62-66,68]
Focus group or group discussion (HSP^b^)	3 (10)	[40-43,53-55]
Interview (HSU)	10 (33)	[26,31,32,34,39,44,45,49,50,59,68]
Interview (HSP)	2 (7)	[28,29,37,38,47]
Design workshop (HSU only)	3 (10)	[30,33,56]
Design workshop (HSP only)	1 (3)	[57]
Co-design workshop (HSU + HSP)	4 (13)	[26,34,51,53-55]
Design activity; for example, card sorting, sketching, affinity wall	7 (23)	[26,30,34,51,56,57,68]
Content creation (HSU)	7 (23)	[26,30,37,38,47,49,51,62,68]
Iterative design process	22 (73)	[26,28,29,31-41,44-47,49-57,60-66]
Prototype, mockup or storyboard	26 (87)	[26,27,30-50,52-59,62-67,69]
Translation	1 (3)	[35,36]
Testing/evaluating prototype	14 (47)	[26,30,31,33,34,39-45,51,56,58,67-69]
Prototype demonstration	2 (7)	[37,38,40,41,47]
Observation	5 (17)	[33,49,53-56,68]
Think aloud	9 (30)	[34,37,38,45-47,49,50,52,60-62]
Screen capture or recording	3 (10)	[33,34,52]
Readability pr health literacy evaluation	2 (7)	[45,49]
Usability testing	8 (27)	[33,34,37,38,45-47,49,57,62]
Cognitive walkthrough	3 (10)	[37,38,46,47,51]
Survey/questionnaire (HSU)	7 (23)	[31,34,39,44,45,48,56,57]
Usability questionnaire	3 (10)	[34,52,57]
Heuristic evaluation	3 (10)	[52,57,60,61]
Thematic or data analysis	14 (47)	[26,28,29,37,38,42,43,45-47,49-57,59]

^a^HSU: health service users.

^b^HSP: health service providers.

**Table 12:** Methods used in stage 4 (operationalization) of CeHRes Roadmap—top 30 studies.

Process/method	Total studies (n=30), n (%)	References
Advisory group involved (HSU^a^)	4 (13)	[30,39,44,60,61,67,69]
Design reflects clinical pathway	1 (3)	[58]
Implementation plan	2 (7)	[37,38,47,60,61]
Feasibility study	2 (7)	[37,38,47,67,69]
Promotional campaign	2 (7)	[30,39,44]
HSU as cofacilitators	1 (3)	[67,69]
Launch event	3 (10)	[35,36,39,44,48]

^a^HSU: health service users.

**Table 13:** Methods used in stage 5 (summative evaluation) of CeHRes Roadmap—top 30 studies.

Process/method	Total studies (n=30), n (%)	References
Test of final version	1 (3)	[33]
Evaluation questionnaire	1 (3)	[35,36]
Usability questionnaire	1 (3)	[33]
Pilot testing	1 (3)	[45]
Randomized controlled trial	10 (33)	[27,33,45,49,50,59-66,68]
Usage statistics and Google Analytics	2 (7)	[39,44,48]
Feedback form, Web survey or follow-up survey	3 (10)	[39,44,48,68]

## Discussion

### Overview

In the era of digital health, we have a plethora of literature describing the need for better engagement with HSU to improve health care and health services, and we have access to the technologies to create a broad array of websites and mobile apps, but we lack detailed protocols for designing eHealth resources. This systematic review explored the participatory methods and frameworks used to engage HSU in the development of eHealth resources throughout the design process. UCD was most commonly reported but varied in its application and intention. Participatory methods promoting HSU engagement ranged from brief consultation via a review process to genuine collaboration, which included additional responsibility for the HSU in the actual creation process. Research and development projects that describe a conceptual model (such as Social Cognitive Theory) and a structured framework (such as the CeHRes Roadmap, which includes a diagram/flowchart) lay the foundations for us to gain greater insight into how particular processes lead to efficacious and effective eHealth resources.

### Electronic Health Initiatives Developed and the Characteristics of Health Service Users

There have been extensive eHealth initiatives to address the issues of accessibility, engagement, health literacy, data collection, health promotion, early intervention, motivation, and behavioral change. Of the 90 MMAT-scored studies, websites and mobile apps make up the majority of eHealth initiatives presented in this review (Multimedia Appendix 5) with a strong multicultural focus (Multimedia Appendix 6). The end users of these eHealth initiatives were young adults, women, and the elderly (Multimedia Appendix 6) with the focus on cancer and mental health (Multimedia Appendix 4). The app has become an engagement tool used by HSP to make health information and health planning more interactive, interesting, and fun for HSU [30,32,35,36,42,43,56]. Moreover, participatory design is thought to enable young people to be creative and have substantial input into the resource development [30].

### Participatory Frameworks

Analyzing the procedural frameworks used in our included studies, we found that no 2 studies reported their processes in the same way. The frameworks governing consumer participation were varied with the most reported being UCD, PAR Framework, CeHRes Roadmap, and MRC Guide to Developing and Evaluating Complex Interventions (Multimedia Appendix 11). The methods implemented to seek the HSU perspectives were also varied with the most reported being focus groups, surveys, interviews, prototype/storyboards, think aloud, and literature search (Tables 9-13). Theories and models that influenced procedures most commonly included cognition, behaviors, processes of change, motivation, and empowerment (Multimedia Appendix 10).

The diversity in eHealth initiatives supports creativity, and to ensure validity and strengthen eHealth research, there is a need to integrate a set of protocols for HSU participation and reporting guidelines [154] available via the Enhancing the QUAlity and Transparency Of health Research Network. This would not constrain methodological innovation and would allow a more effective meta-analysis and comparison of participatory development studies.

### Methods Used in the Development of Resources

This review looked for evidence of sound methods for engaging HSU during the development of eHealth apps, tools, and resources. We found relatively few reports that described HSU participation throughout development (ie, from contextual enquiry to summative evaluation, Table 5). Furthermore, many of these reports did not provide adequate details according to mixed-methods appraisal standards. As shown in Multimedia Appendix 2, studies out of 603 full texts reviewed met all of our inclusion criteria and scored 90% or higher according to MMAT. This suggests that research training, funding, and dissemination agencies need to attach far greater importance to reports that describe methods more rigorously.

Others have observed that “The diverse communities working in digital health—including government stakeholders, technologists, clinicians, implementers, network operators, researchers, donors—have lacked a mutually understandable language with which to assess and articulate functionality” [155]. Tables 9-13 illustrates how deeply this lack has affected the production of cohesive research evidence, that is, it is virtually impossible to map the semantic relationships among the methodology elements to inform the discourse about what forms of participatory eHealth design work and why. Many methods are generic to human computer interaction, some take a broad behavioral approach and some include methods of measuring health outcomes in the particular area of health where the intervention is directed. One possible view is that this illustrates a flourishing of innovation and creativity. Another is that this creates a minefield for research training and peer reviewing and may represent a considerable waste of research resources.

Analyzing the conceptual bases for the methods used in the 30 studies scoring 90% or higher on MMAT, we found much variety with 23 different models or theories reported (Multimedia Appendix 10). The most commonly occurring theories were Social Cognitive Theory, Theory of Planned Behavior, Transtheoretical Model, Persuasive Technology Theory, and Health Behavior Theory. This finding offers a sound basis in evidence for future researchers who wish to follow these precedents. However, we note that research in this area has not been informed by other potentially relevant theories (for example, theories that may account better for healthcare consumers’ economic, emotional, or empowerment motives for engagement) [156].

### Effective Involvement of Health Service Users

This review looked for evidence about the effectiveness of particular approaches in terms of supporting involvement by HSU. Winterling reported strategies implemented to address engagement with HSU, including 1-person central contact, established expectation of roles, compensation for time, reaching a common agreement, and HSU seen as experts on patient perspective [63-66].

It is also possible to reflect on the richness of the findings generated by particular approaches. As shown in Multimedia Appendix 13, each study reported between 2 and 10 major thematic outputs. Reports with relatively concise outputs were Bengtsson [28,29] using participatory research design and O’Brien [51] describing an array of approaches. The most extensive review was reported by Fleisher [68] using the Ottawa Decision Support Framework and participatory design and Goldenberg [42,43] using 3 types of iterative qualitative research approaches. In assessing effectiveness this way, unknown factors may be in play, such as sophistication of the data collection procedures, analytical expertise of the researchers, editorial constraints on reporting results, and temporal pressures on publication.

### Important Aspects of Participatory Methods for Health Service Users

There were consistent themes that represented HSU priorities in eHealth initiatives across the selected 30 studies represented in Multimedia Appendix 13. Access to relevant, simple, and clear health information was reported consistently across most of the studies highlighting the importance of this information to make informed decisions in a timely manner. A well-designed eHealth resource that includes a framework supporting HSU involvement can significantly impact health literacy for both HSU and HSP. HSU involvement with the development of an eHealth resource created a collaborative process that required transparency and respect as well as clear mediation processes [53-55].

Being involved in the development of an eHealth resource created the opportunity for HSU to clarify the user perspective and support the relevance of the final product. Despite the variety of websites and apps, HSU reported the need for improved access to information, coordination of care, interactivity with information provided, culturally specific information, patient education, and self-management. HSU also acknowledged the importance of confidentiality and privacy when exchanging personal health information over electronic networks.

### Impact of Participatory Methods Reported by Researchers

The researchers reported a number of key issues highlighting the importance of participatory methods in creating an eHealth resource that was relevant to HSU. In Multimedia Appendix 13, an outline of the research recommendations was documented for the selected 30 studies. Researchers reported on the importance of utilizing a participatory design, which included an iterative process that increased the responsiveness and relevance of their eHealth initiatives. Having the HSU perspective from the beginning was important as well as ensuring that the process was genuinely collaborative with all participants respected and acknowledged. Utilizing a health behavior theory in combination with a participatory design was noted to enhance the eHealth resource. The theory base acknowledges the importance of motivation, empowerment, and stages of change in supporting the engagement and utilization of the eHealth resource. It was also noted that the eHealth resource needed to be interesting, engaging, and in some instances include a game-playing element. Creating a more positive approach enabled the HSU to engage with serious and difficult health issues and explore options for improved health. Not only did the eHealth resource need to be interesting but it also importantly needed to be intuitive and simple to navigate.

Heckman [45] reported that their eHealth initiative was guided by intervention development, assessment guidelines for behavioral therapy, and health communication programs with health literacy best-practice. Utilizing a participatory design appeared to improve the relevance of the eHealth resource by addressing issues of culture, gender, age, and sexuality (Multimedia Appendix 6). Goldenberg [42,43] reported personalization along with interactive functionality promoted ownership for HSU. A majority of projects included both HSU and HSP in participatory methods across different developmental stages from contextual inquiry to summative evaluation of the project [28,29,31,34-38,40-43,45-55,58-61,63-67,69]. Evaluation is an integral part of participatory methodology; however, this was reported inconsistently across the 30 studies (Multimedia Appendix 8). The inclusion of a standardized tool to evaluate processes and outcomes from the HSU perspective, as part of a participatory framework, may address the need to bring more objectivity to evaluating various studies.

The demand on time and financial resources to implement a participatory design was noted by some researchers [27,30,42,43,57,68]. Availability of resources was an important consideration throughout the design process, which was often iterative. With the rapid change in technology, there is an increasing demand for HSP to be agile and develop eHealth resources more quickly but still maintaining an evidence-based, best-practice approach inclusive of HSU participation.

### Limitations

A limitation of our final dataset is that because of the number of papers retrieved, we decided to limit our analysis to published journal articles and to leave out full papers in conference proceedings. It is possible that there are strong participatory processes that have not been reported in detail, or at all, in the journal literature. Moreover, we did not include studies published in languages other than English and therefore we cannot be certain that our dataset reflects work being done around the world.

As our focus was on the inclusion of HSU from the early development process onward, some studies were included that did not extend to a final evaluation of the product, and it was not always possible to consider the success or otherwise of the final eHealth product. As a part of our inclusion criteria, we required some evidence that a specific eHealth product was ultimately created or likely to be taken to completion.

A limitation of our data analysis is that MMAT is a critical appraisal tool to assess the methodological quality of studies. It does not assess the quality of the writing or the content of the research; therefore, it is possible that we have overlooked papers that may be of high quality in other respects but which we have not rated highly here because of the way their methods sections are presented. For example, under MMAT, a paper will not score highly if it does not discuss the impact of the research or report the limitations of a mixed-methods study. The studies may not have rated highly under MMAT if they used both qualitative and quantitative methods but did not acknowledge that this constituted a mixed-methods study or if only selected aspects were reported. For example, a study that reported HSU participation only at the summative evaluation stage may have involved HSU earlier as well, but this would not register in our search results because we looked for descriptions of methods for HSU participation from initial design stages.

Although categorizing all reported methods in these studies according to the 5 stages of the CeHRes Roadmap [25] was a generally useful way to compare processes across studies, absolute consistency was not achievable because of the wide variety of structuring reports, the differing terminology and naming conventions used for similar methods, and the difficulty in allocating all methods accurately to a particular process stage.

### Conclusions

Agility of eHealth development is problematic in comparison to nonmedical industries as we seek to ensure safety and quality of care for HSU. It is a challenge for eHealth development to follow rigorous methods within a timeframe that responds to current needs, limited resources, and rapid technological changes. Methodological approaches to developing eHealth resources vary but the importance of engaging HSU in participatory design is consistently emphasized. By synthesizing the existing evidence about strong mixed methods for participatory development of eHealth resources, we anticipate that this systematic review will provide others with clearer guidance to plan more rapid and better-structured work of this kind.

## Appendix

Multimedia Appendix 1 Search strategies for all databases.

Multimedia Appendix 2 Scoring of 90 studies according to the Mixed Methods Appraisal Tool Version 2011.

Multimedia Appendix 3 Descriptive summary of 90 studies.

Multimedia Appendix 4 Major health focus in 90 studies.

Multimedia Appendix 5 Electronic health technology developed in 90 studies.

Multimedia Appendix 6 Targeted populations in 90 studies.

Multimedia Appendix 7 Countries where study took place in 90 studies.

Multimedia Appendix 8 Detail of data extracted from 30 studies.

Multimedia Appendix 9 Health area and purpose of product in top 30 studies categorised using Medical Subject Headings (MeSH).

Multimedia Appendix 10 Model or theory base in top 30 studies.

Multimedia Appendix 11 Participatory frameworks and approaches in top 30 studies.

Multimedia Appendix 12 Interventions in top 30 studies.

Multimedia Appendix 13 Themes and recommendations in top 30 studies.

## Abbreviations

ACM: Association for Computing Machinery
CeHRes: Center for eHealth Research and Disease Management
CINAHL: Cumulative Index to Nursing and Allied Health Literature
eHealth: electronic health
HSP: health service providers
HSU: health service users
IEEE: Institute of Electrical and Electronics Engineers
MeSH: Medical Subject Headings
MMAT: Mixed Methods Appraisal Tool
MRC: Medical Research Council
OHP: Optimal Health Program
PAR: participatory action research
UCD: User-Centered Design
