# Supplementary material for: Participatory Methods to Engage Health Service Users in the Development of Electronic Health Resources: Systematic Review
Source: J Particip Med. 2019 Feb 22;11(1):e11474. doi: 10.2196/11474 (PMC7434099; doi:10.2196/11474)
Supplement: Multimedia Appendix 1 [file jopm_v11i1e11474_app1.pdf]

Ovid MEDLINE(R) Epub Ahead of Print, In-Process & Other Non-Indexed Citations, Ovid MEDLINE(R) Daily and Ovid MEDLINE(R) 1946 to Present

Last searched: 1 February 2017

1. Community-Based Participatory Research/ or consumer participation/ or patient participation/
2. (codesign* or co-design* or coproduc* or co-produc* or cocreat* or co-creat* or participatory or e-collaboration or usability or focus group*).ti,ab.
3. ((user* or patient* or consumer* or family or families or carer* or caregiver* or participant* or client* or stakeholder* or peer*) adj2 (centre* or center* or centric or involv* or participat* or partner* or activat* or experience or advisor* or includ* or inclusion or engag* or collaborat* or consult* or empower* or input* or led or focus*)).ti,ab.
4. 1 or 2 or 3
5. internet/ or blogging/ or social media/ or audiovisual aids/ or multimedia/ or cell phones/ or text messaging/ or webcasts as topic/ or Telemedicine/ or videoconferencing/ or educational technology/ or motion pictures as topic/ or exp optical storage devices/ or radio/ or exp tape recording/ or exp television/ or Mobile Applications/ or Software Design/
6. (internet or web* or online or www* or audiovisual* or audio-visual* or multimedia or multi-media or ehealth or e-health or mobile tech* or mobile phone* or mobile device* or mobile health or mhealth or m-health or cell phone or cellular phone or smartphone or app or apps or blog* or social media or social network* or facebook or podcast* or tracking device* or electronic health device* or fitbit or elearning or e-learning or wearable device* or smartwatch* or wearable electronics or telemedicine or tele-medicine or telehealth or tele-health or video* or electronic patient record* or electronic medical record* or electronic health record* or electronic record* or wiki* or portal* or behavioral intervention technolog* or health information technolog* or software or medical informatic* or health informatic* or digital health).ti.
7. 5 or 6
8. Program development/ or planning techniques/ or equipment Design/ or software design/
9. (develop* or creat* or plan* or build* or implement* or codesign* or co-design*).ti,ab. or design*.ti.
10. 8 or 9
11. 4 and 7 and 10
12. limit 11 to (english language and yr="2006 -2016")

EMBASE (Embase.com)

Last searched: 1 February 2017

#1 'consumer'/mj or 'participatory research'/de or 'participatory management'/de

#2 codesign*:ab,ti or co-design*:ab,ti or coproduc*:ab,ti or co-produc*:ab,ti or participatory:ab,ti or 'e collaboration':ab,ti or usability:ab,ti or 'focus group':ab,ti OR 'focus groups':ab,ti

#3 ((user* OR patient* OR consumer* OR family OR families OR carer* OR caregiver* OR participant* OR client* OR stakeholder* OR peer*) NEAR/2 (centre* OR center* OR centric OR involv* OR participat* OR partner* OR activat* OR experience OR advisor* OR includ* OR inclusion OR engag* OR collaborat* OR consult* OR empower* OR input* OR led OR focus*)):ab,ti

#4 #1 or #2 or #3

#5 'internet'/mj or 'social media'/mj or 'audiovisual aid'/mj or 'telemedicine'/mj OR 'telehealth'/mj or 'mobile phone'/mj or 'text messaging'/mj OR 'videoconferencing'/mj OR 'webcast'/mj or 'audiovisual equipment'/mj OR 'multimedia'/mj OR 'optical disk'/mj OR 'television'/mj OR 'video disk'/mj OR 'videotape'/mj OR ‘mobile application’/mj

#6 internet:ti OR web*:ti OR online:ti OR www*:ti OR audiovisual*:ti OR 'audio visual':ti OR multimedia:ti OR 'multi-media':ti OR ehealth:ti OR 'e-health':ti OR 'mobile technology':ti OR 'mobile phone':ti OR 'mobile device':ti OR 'mobile health':ti OR mhealth:ti OR 'm-health':ti OR 'cell phone':ti OR 'cellular phone':ti OR smartphone:ti OR app:ti OR apps:ti OR ‘mobile application’:ti OR blog*:ti OR 'social media':ti OR 'social network':ti OR facebook:ti OR podcast*:ti OR 'tracking device':ti OR 'electronic health device':ti OR fitbit:ti OR elearning:ti OR 'e-learning':ti OR 'wearable device':ti OR smartwatch*:ti OR 'wearable electronics':ti OR telemedicine:ti OR 'tele-medicine':ti OR telehealth:ti OR 'tele-health':ti OR video*:ti OR 'electronic patient

record':ti OR 'electronic medical record':ti OR 'electronic health record':ti OR 'electronic record':ti OR wiki*:ti OR portal*:ti OR 'behavioral intervention technology':ti OR 'behavioural intervention technologies':ti OR 'medical informatics':ti OR 'health informatics':ti OR ‘digital health’:ti

#7 #5 or #6

#8 'planning'/de OR 'health care planning'/de OR 'program development'/de OR 'equipment design'/de

#9 develop*:ab,ti OR creat*:ab,ti OR plan*:ab,ti OR build*:ab,ti OR implement*:ab,ti OR codesign*:ab,ti OR co-design*:ab,ti OR design*:ti

#10 #8 or #9

#11 #4 and #7 and #10

#12 #4 and #7 and #10 AND [english]/lim AND [2006-2016]/py

CINAHL Plus with Full Text (EBSCOhost)

Last searched: 1 February 2017

S1 (MH "Consumer Participation")

S2 codesign* or co-design* or coproduc* or co-produc* or cocreat* or co-creat* or participatory or e-collaboration or usability or focus group*

S3 (user* or patient* or consumer* or family or families or carer* or caregiver* or participant* or client* or stakeholder* or peer*) N2 (centre* or center* or centric or involv* or participat* or partner* or activat* or experience or advisor* or includ* or inclusion or engag* or collaborat* or consult* or empower* or input* or led or focus*)

S4 S1 OR S2 OR S3

S5 TI internet or TI web* or TI online or TI www* or TI audiovisual* or TI audio-visual* or TI multimedia or TI multi-media or TI ehealth or TI e-health or TI mobile tech* or TI mobile phone* or TI mobile device* or TI mobile health or TI mhealth or TI m-health or TI cell phone or TI cellular phone or TI smartphone or TI mobile application* or TI app or TI apps or TI blog* or TI social media or TI social network* or TI facebook or TI podcast* or TI tracking device* or TI electronic health device* or TI fitbit or TI elearning or TI e-learning or TI wearable device* or TI smartwatch* or TI wearable electronics or TI telemedicine or TI tele-medicine or TI telehealth or TI tele-health or TI video* or TI electronic patient record* or TI electronic medical record* or TI electronic health record* or TI electronic record* or TI wiki* or TI portal* or TI behavioral intervention technolog* or TI health information technolog* or TI software or TI medical informatic* or TI health informatic* or TI digital health

S6 (MM "Internet+") OR (MM "Electronic Bulletin Boards+") OR (MM "Teleconferencing") OR (MM "Telehealth+") OR (MM "Television") OR (MM "Text Messaging") OR (MM "Videoconferencing+") OR (MM "Audiovisuals") OR (MM "Audiorecording") OR (MM "Videorecording+") OR (MM "Multimedia") OR (MM "Mobile Applications")

S7 S5 OR S6

S8 (MH "Program Development") OR (MH "Program Evaluation") OR (MH "Program Implementation") OR (MH "Planning Techniques") OR (MH "Program Planning") OR (MH "Equipment Design") OR (MH "Software Design")

S9 develop* or creat* or plan* or build* or implement* or codesign* or co-design* or TI design*

S10 S8 OR S9

S11 S4 AND S7 AND S10 Limiters - Published Date: 20060101-20161231; English Language

PsycINFO 1806 to January Week 4 2017

Last searched: 1 February 2017

1. client participation/
2. (codesign* or co-design* or coproduc* or co-produc* or cocreat* or co-creat* or participatory or e-collaboration or usability or focus group*).ti,ab.
3. ((user* or patient* or consumer* or family or families or carer* or caregiver* or participant* or client* or stakeholder* or peer*) adj2 (centre* or center* or centric or involv* or participat* or partner* or activat* or experience or advisor* or includ* or inclusion or engag* or collaborat* or consult* or empower* or input* or led or focus*)).ti,ab.
4. 1 or 2 or 3
5. internet/ or online therapy/ or social media/ or telemedicine/ or telecommunications media/ or websites/ or audiovisual communications media/ or multimedia/ or audiotapes/ or digital video/ or educational audiovisual aids/ or films/ or television/ or videotapes/ or cellular phones/ or mobile devices/
6. (internet or web* or online or www* or audiovisual* or audio-visual* or multimedia or multi-media or ehealth or e-health or mobile tech* or mobile phone* or mobile device* or mobile health or mhealth or m-health or cell phone or cellular phone or smartphone or app or apps or blog* or social media or social network* or facebook or podcast* or tracking device* or electronic health device* or fitbit or elearning or e-learning or wearable device* or smartwatch* or wearable electronics or telemedicine or tele-medicine or telehealth or tele-health or video* or electronic patient record* or electronic medical record* or electronic health record* or electronic record* or wiki* or portal* or behavioral intervention technolog* or health information technolog* or medical informatic* or health informatic* or software or digital health).ti.
7. 5 or 6
8. program development/ or educational program planning/ or product design/
9. (develop* or creat* or plan* or build* or implement* or codesign* or co-design*).ti,ab. or design*.ti.
10. 8 or 9
11. 4 and 7 and 10
12. limit 11 to (english language and yr="2006 -2016")

Ovid Emcare 1995 to 2016 week 49

Last searched: 1 February 2017

1. patient participation/ or participatory research/ or participatory management/
2. (codesign* or co-design* or coproduc* or co-produc* or cocreat* or co-creat* or participatory or e-collaboration or usability or focus group*).ti,ab.
3. ((user* or patient* or consumer* or family or families or carer* or caregiver* or participant* or client* or stakeholder* or peer*) adj2 (centre* or center* or centric or involv* or participat* or partner* or activat* or experience or advisor* or includ* or inclusion or engag* or collaborat* or consult* or empower* or input* or led or focus*)).ti,ab.
4. 1 or 2 or 3
5. internet/ or blogging/ or social media/ or audiovisual aid/ or multimedia/ or exp mobile phone/ or text messaging/ or webcast/ or telemedicine/ or videoconferencing/ or educational technology/ or movie/ or mobile application/ or software design/
6. (internet or web* or online or www* or audiovisual* or audio-visual* or multimedia or multi-media or ehealth or e-health or mobile tech* or mobile phone* or mobile device* or mobile health or mhealth or m-health or cell phone or cellular phone or smartphone or app or apps or blog* or social media or social network* or facebook or podcast* or tracking device* or electronic health device* or fitbit or elearning or e-learning or wearable device* or smartwatch* or wearable electronics or telemedicine or tele-medicine or telehealth or tele-health or video* or electronic patient record* or electronic medical record* or electronic health record* or electronic record* or wiki* or portal* or behavioral intervention technolog* or health information technolog* or software or medical informatic* or health informatic* or digital health).ti.
7. 5 or 6
8. program development/ or planning/ or equipment design/ or software design/

9. (develop* or creat* or plan* or build* or implement* or codesign* or co-design*).ti,ab. or design*.ti.
10. 8 or 9
11. 4 and 7 and 10
12. limit 11 to (english language and yr="2006 - 2016")

Cochrane Library

Last searched: 1 February 2017

- Cochrane Database of Systematic Reviews : Issue 1 of 12, January 2017
- Database of Abstracts of Reviews of Effect : Issue 2 of 4, April 2015
- Cochrane Central Register of Controlled Trials : Issue 11 of 12, November 2016
- Cochrane Methodology Register : Issue 3 of 4, July 2012
- Health Technology Assessment Database : Issue 4 of 4, October 2016
- NHS Economic Evaluation Database : Issue 2 of 4, April 2015
- About the Cochrane Collaboration : Issue 1 of 12, January 2017

#1 [mh "Community-Based Participatory Research"] or [mh "consumer participation"] or [mh "patient participation"]
#2 codesign*:ti or co-design*:ti or coproduc*:ti or co-produc*:ti or participatory:ti or e-collaboration:ti or usability:ti or focus group*:ti
#3 ((user* or patient* or consumer* or family or families or carer* or caregiver* or participant* or client* or stakeholder* or peer*) near/2 (centre* or center* or centric or involv* or participat* or partner* or activat* or experience or advisor* or includ* or inclusion or engag* or collaborat* or consult* or empower* or input* or led or focus*)):ti
#4 #1 or #2 or #3
#5 [mh internet] or [mh blogging] or [mh "social media"] or [mh "audiovisual aids"] or [mh multimedia] or [mh "cell phones"] or [mh "text messaging"] or [mh "webcasts as topic"] or [mh telemedicine] or [mh videoconferencing] or [mh "educational technology"] or [mh "audiovisual aids"] or [mh "motion pictures as topic"] or [mh multimedia] or [mh "optical storage devices"] or [mh radio] or [mh "tape recording"] or [mh television] or [mh "mobile applications"] or [mh "software design"]
#6 (internet or web* or online or www* or audiovisual* or audio-visual* or multimedia or multi-media or ehealth or e-health or mobile tech* or mobile phone* or mobile device* or mobile health or mobile application* or mhealth or m-health or cell phone or cellular phone or smartphone or app or apps or blog* or social media or social network* or facebook or podcast* or tracking device* or electronic health device* or fitbit or elearning or e-learning or wearable device* or smartwatch* or wearable electronics or telemedicine or tele-medicine or telehealth or tele-health or video* or electronic patient record* or electronic medical record* or electronic health record* or electronic record* or wiki* or portal* or behavioral intervention technolog* or health information technolog* or software or medical informatics* or health informatics* or digital health):ti
#7 #5 or #6
#8 #4 and #7 (Publication Year from 2006 to 2016)

Web of Science Core Collection

Last searched: 15 February 2017

Indexes=SCI-EXPANDED, SSCI, A&HCI, CPCI-S, CPCI-SSH, BKCI-S, BKCI-SSH, ESCI, CCR-EXPANDED, IC Timespan=2006-2016

Web of Science Core Collection: Citation Indexes
Science Citation Index Expanded (SCI-EXPANDED) --1900-present
Social Sciences Citation Index (SSCI) --1900-present
Arts & Humanities Citation Index (A&HCI) --1975-present
Conference Proceedings Citation Index- Science (CPCI-S) --1990-present
Conference Proceedings Citation Index- Social Science & Humanities (CPCI-SSH) --1990-present
Book Citation Index– Science (BKCI-S) --2005-present

Book Citation Index– Social Sciences & Humanities (BKCI-SSH) --2005-present
Emerging Sources Citation Index (ESCI) --2015-present

#1 (WC=(Behavioral Sciences or Health Care Sciences & Services or Health Policy & Services or Medical Informatics or Medicine, General & Internal or Nursing or Nutrition & Dietetics or Obstetrics & Gynecology or Oncology or Orthopedics or Pediatrics or Primary Health Care or Psychiatry or Psychology or Rehabilitation)) AND LANGUAGE: (English)
#2 (TS=(health or medical or illness or wellbeing or patient or hospital)) AND LANGUAGE: (English)
#3 #1 OR #2
#4 (TI=(internet or web or online or www or audiovisual or audio-visual or multimedia or multi-media or ehealth or e-health or mobile technology or mobile phone* or mobile device* or mobile health or mhealth or m-health or cell phone or cellular phone or smartphone or "app" or "apps" or mobile application* or blog* or social media or facebook or podcast* or tracking device* or electronic health device* or fitbit or elearning or e-learning or wearable device* or smartwatch or wearable electronics or telemedicine or tele-medicine or telehealth or tele-health or video or electronic patient record* or electronic medical record* or electronic health record* or electronic record* or wiki* or portal* or behavioural intervention technolog* or health information technolog* or software or medical informatics* or health informatics* or digital health)) AND LANGUAGE: (English)
#5 (WC=Medical Informatics) AND LANGUAGE: (English)
#6 #4 OR #5
#7 (TI=(develop* or creat* or plan* or build* or implement* or codesign* or co-design* or design*)) AND LANGUAGE: (English)
#8 (TI=(codesign* or co-design* or coproduc* or co-produc* or cocreat* or co-creat* or participat* or collaborat* or focus group* or user* or stakeholder* or consumer* or client* or "centered" or "centred" or "centric")) AND LANGUAGE: (English)
#9 #8 AND #7 AND #6 AND #3
#10 Limited results to articles, proceedings papers, reviews and corrections, 2006-2016, English language

ACM Guide to Computing Literature

Last searched: 15 February 2017

"query": { ("health" "illness" "wellbeing" “patient” “hospital” “medical” “ehealth” “mhealth”)
AND acmdlTitle:("codesign" "co-design" “coproduc” “co-produc” "participation" "participatory" "centered" "centred" "centric" "focus group" “stakeholder” "user" "consumer")
AND acmdlTitle:("internet" "blog" "social media" "audiovisual" "multimedia" "cell phone" "text messaging" "web" "telemedicine" "video" "technology" "mobile" "application" "app" "software" "informatic" "www" "ehealth" "mhealth" "smartphone" "facebook" "podcast" "tracking device" "wearable device" "smartwatch" "telemedicine" "telehealth" "electronic patient record" "electronic medical record" "electronic health record" "electronic record" "wiki" "portal" "informatic")
AND acmdlTitle:("develop" "design" "plan" "create" “codesign” “co-design” "build" "implement") }

"filter": {"publicationYear":{ "gte":2006 }},
{owners.owner=GUIDE}

IEEE Xplore Digital Library

Last searched: 7 February 2017

#1 health OR ehealth OR mhealth OR medical OR illness OR wellbeing OR patient OR hospital
#2 “Document Title”:codesign* OR Document Title:co-design* OR Document Title:participatory OR Document Title:participation OR Document Title:user* OR Document Title:stakeholder OR Document Title:focus group OR “Document Title":consumer OR “Document Title":patient OR “Document Title":client
#3 #1 and #2
#4 Results limited to Conference publications, Journals and magazines, early access articles and 2006 to 2016
