# Supplementary material for: Participatory Methods to Engage Health Service Users in the Development of Electronic Health Resources: Systematic Review
Source: J Particip Med. 2019 Feb 22;11(1):e11474. doi: 10.2196/11474 (PMC7434099; doi:10.2196/11474)
Supplement: Multimedia Appendix 2 [file jopm_v11i1e11474_app2.pdf]

Reference for Mixed Methods Appraisal Tool (MMAT): Pluye P, Robert E, Cargo M, Bartlett G, O'Cathain A, Griffiths F, et al. Proposal: A mixed methods appraisal tool for systematic mixed studies reviews. . Department of Family Medicine, McGill University, Montreal, Canada2011 [cited 2019 20 Jan]; Available from: http://mixedmethodsappraisaltoolpublic.pbworks.com/w/file/fetch/84371689/MMAT%202011%20criteria%20and%20tutorial%202011-06-29updated2014.08.21.pdf Archived by WebCite® at http://www.webcitation.org/75U179AyW.

Three ratings are possible under each criteria: Y = YES (green); N = NO (red); C = CAN'T TELL (yellow)

Study / References	MMAT score %	Study type	Qual 1.1	Qual 1.2	Qual 1.3	Qual 1.4	RCT 2.1	RCT 2.2	RCT 2.3	RCT 2.4	Non RCT 3.1	Non RCT 3.2	Non RCT 3.3	Non RCT 3.4	Desc 4.1	Desc 4.2	Desc 4.3	Desc 4.4	Mix 5.1	Mix 5.2	Mix 5.3
Ahtinen 2013 [26]	100	Qual	Y	Y	Y	Y															
Antypas 2014 [27]	100	Qual	Y	Y	Y	Y															
Bengtsson 2014 [28, 29]	100	Mixed	Y	Y	Y	Y									Y	Y	Y	Y	Y	Y	Y
Buccieri 2015 [30]	100	Qual	Y	Y	Y	Y															
Clayman 2008 [31]	100	Qual	Y	Y	Y	Y															
Cordova 2015 [32]	100	Qual	Y	Y	Y	Y															
Dabbs 2009 [33]	100	Mixed	Y	Y	Y	Y									Y	Y	Y	Y	Y	Y	Y
Das 2013 [34]	100	Qual	Y	Y	Y	Y															
Davies 2015 [35, 36]	100	Mixed	Y	Y	Y	Y									Y	Y	Y	Y	Y	Y	Y
Fennell 2016 [39, 44]	100	Mixed	Y	Y	Y	Y									Y	Y	Y	Y	Y	Y	Y
Fonda 2010 [40, 41]	100	Qual	y	Y	Y	Y															
Goldenberg 2015 [42, 43]	100	Mixed	Y	Y	Y	Y									Y	Y	Y	Y	Y	Y	y
Heckman 2015 [45]	100	Mixed	Y	Y	Y	Y									Y	Y	Y	Y	Y	Y	Y
Kelders 2013 [46]	100	Mixed	Y	Y	Y	Y									Y	Y	Y	Y	Y	Y	Y
Lubberding 2015 [37, 38, 47]	100	Mixed	Y	Y	Y	Y									Y	Y	Y	Y	Y	Y	Y
Meyer 2007 [48]	100	Qual	Y	Y	Y	Y															
Miller 2015 [49]	100	Mixed	Y	Y	Y	Y									Y	Y	Y	Y	Y	Y	Y
Morrison 2015 [50]	100	Qual	Y	Y	Y	Y															
O'Brien 2016 [51]	100	Qual	Y	Y	Y	Y															
Peute 2015 [52]	100	Mixed	Y	Y	Y	Y									Y	Y	Y	Y	Y	Y	Y
Revenas 2015 [53-55]	100	Qual	Y	Y	Y	Y															
Sandlund 2015 [56]	100	Qual	Y	Y	Y	Y															
Schnall 2014 [57]	100	Mixed	Y	Y	Y	Y									Y	Y	Y	Y	Y	Y	Y

Study / References	MMAT score %	Study type	Qual 1.1	Qual 1.2	Qual 1.3	Qual 1.4	RCT 2.1	RCT 2.2	RCT 2.3	RCT 2.4	Non RCT 3.1	Non RCT 3.2	Non RCT 3.3	Non RCT 3.4	Desc 4.1	Desc 4.2	Desc 4.3	Desc 4.4	Mix 5.1	Mix 5.2	Mix 5.3
Skjoth 2015 [58]	100	Qual	Y	Y	Y	Y															
Stinson 2014 [59]	100	Mixed	Y	Y	Y	Y									Y	Y	Y	Y	Y	Y	Y
van Bruinessen 2014 [60, 61]	100	Qual	Y	Y	Y	Y															
Widman 2016 [62]	100	Qual	Y	Y	Y	Y															
Winterling 2016 [63-66]	100	Qual	Y	Y	Y	Y															
Ennis 2014 [67, 69]	90	Mixed	Y	Y	Y	Y									Y	Y	Y	Y	Y	Y	N
Fleisher 2014 [68]	90	Mixed	Y	Y	Y	Y									Y	Y	Y	Y	Y	Y	N
Alnasser 2016 [70,71]	75	Mixed	Y	Y	Y	N									Y	Y	Y	Y	Y	Y	N
Alvarado-Martel 2015 [72]	75	Mixed	Y	Y	Y	N									Y	Y	Y	N	Y	Y	N
Andersen 2011 [74]	75	Qual	N	Y	Y	Y															
Armstrong 2007 [75-77]	75	Qual	Y	Y	Y	N															
Atkinson 2009 [78]	75	Mixed	Y	Y	Y	N									Y	Y	Y	Y	Y	Y	N
Bae 2009 [79]	75	Mixed	Y	Y	Y	N									Y	Y	Y	N	Y	Y	N
Boyd 2015 [81]	75	Qual	Y	Y	Y	N															
Buckingham 2015 [82]	75	Qual	Y	Y	Y	N															
Cade 2013 [83,85]	75	Mixed	Y	Y	Y	N									Y	Y	Y	C	Y	Y	N
Cafazzo 2012 [84]	75	Mixed	Y	Y	Y	N									Y	Y	Y	Y	Y	Y	N
Cnossen 2015 [86]	75	Qual	Y	Y	Y	N															
Colombo 2016 [87]	75	Mixed	Y	Y	Y	N									Y	Y	Y	Y	Y	Y	N
Coyne 2016 [88]	75	Mixed	Y	Y	Y	N									Y	Y	Y	C	Y	Y	N
Danaher 2012 [89]	75	Mixed	Y	Y	Y	N									Y	Y	Y	C	Y	Y	Y
Davies 2009 [90,123]	75	Qual	Y	Y	Y	N															
Dykes 2014 [91]	75	Qual	Y	Y	Y	N															
Enah 2014 [92,93]	75	Mixed	Y	Y	Y	N									Y	Y	Y	Y	Y	Y	N
Fink 2015 [94]	75	Mixed	Y	Y	Y	N	Y	N	Y	Y									Y	Y	N
Fledderus 2015 [95]	75	Mixed	Y	Y	Y	N									Y	Y	Y	Y	Y	Y	N
Graham 2014 [96]	75	Mixed	Y	Y	Y	N									Y	Y	Y	Y	Y	Y	N
Hearn 2014 [97,98]	75	Mixed	Y	Y	Y	N									Y	Y	Y	Y	Y	Y	N

Study / References	MMAT score %	Study type	Qual 1.1	Qual 1.2	Qual 1.3	Qual 1.4	RCT 2.1	RCT 2.2	RCT 2.3	RCT 2.4	Non RCT 3.1	Non RCT 3.2	Non RCT 3.3	Non RCT 3.4	Desc 4.1	Desc 4.2	Desc 4.3	Desc 4.4	Mix 5.1	Mix 5.2	Mix 5.3
Hightow-Weidman 2011 [99]	75	Qual	Y	Y	Y	N															
Horne 2016 [100]	75	Mixed	Y	Y	Y	Y	Y	N	Y	Y									Y	Y	Y
Kattelmann 2014 [101]	75	Mixed	Y	Y	Y	N									Y	Y	Y	Y	Y	Y	N
Kim 2015 [102]	75	Mixed	Y	Y	Y	N									Y	Y	Y	Y	Y	Y	Y
Kuijpers 2015 [103,104]	75	Mixed	Y	Y	Y	N									Y	Y	Y	Y	Y	Y	N
Lee 2013 [105]	75	Mixed	Y	Y	Y	N									Y	Y	Y	Y	Y	Y	N
Martin-Hammond 2015 [106]	75	Mixed	Y	Y	Y	N									Y	Y	Y	Y	Y	Y	N
Neville 2016 [107,108]	75	Mixed	Y	Y	Y	N									Y	Y	Y	Y	Y	Y	N
Schlosser 2016 [109]	75	Mixed	Y	Y	Y	N									Y	Y	Y	Y	Y	Y	N
Siek 2011 [110]	75	Mixed	Y	Y	Y	Y									Y	Y	Y	Y	Y	Y	N
Todd 2013 [111,122]	75	Mixed	Y	Y	Y	Y	Y	N	Y	Y									Y	Y	N
Trudeau 2011 [112]	75	Mixed	Y	Y	Y	N									Y	N	Y	Y	Y	Y	N
Vonk Noordegraaf 2012 [113]	75	Qual	Y	Y	Y	N															
Ward 2016 [114]	75	Mixed	Y	Y	Y	N									Y	Y	Y	Y	Y	Y	N
Waterlander 2014 [115]	75	Mixed	Y	Y	Y	N									Y	Y	Y	Y	Y	Y	Y
Whitehouse 2013 [116]	75	Mixed	Y	Y	Y	N									Y	Y	Y	Y	Y	Y	N
Whittaker 2008 [117]	75	Mixed	Y	Y	Y	N									Y	Y	Y	Y	Y	Y	N
Williamson 2016 [80,118,119]	75	Qual	Y	Y	Y	N															
Wright 2016 [120]	75	Mixed	Y	Y	Y	N									Y	Y	Y	Y	y	y	y
Wysocki 2016 [121]	75	Qual	y	y	y	n															
Crosby 2016 [124]	50	Mixed	Y	Y	Y	Y									Y	C	Y	C	Y	C	Y
Foster 2015 [125]	50	Qual	Y	Y	N	N															
Gordon 2015 [126]	50	Mixed	Y	N	Y	Y									Y	Y	Y	Y	Y	N	N
Grant 2012 [127]	50	Mixed	Y	Y	N	N									Y	Y	Y	Y	Y	Y	N
Groussard 2015	50	Mixed	Y	C	Y	N									Y	Y	Y	Y	Y	Y	N

Study / References	MMAT score %	Study type	Qual 1.1	Qual 1.2	Qual 1.3	Qual 1.4	RCT 2.1	RCT 2.2	RCT 2.3	RCT 2.4	Non RCT 3.1	Non RCT 3.2	Non RCT 3.3	Non RCT 3.4	Desc 4.1	Desc 4.2	Desc 4.3	Desc 4.4	Mix 5.1	Mix 5.2	Mix 5.3
[128]																					
Hallett 2009 [129]	50	Mixed	Y	Y	Y	N	N	Y	N	N									Y	N	N
Heinrich 2009 [130]	50	Qual	N	Y	Y	N															
McCarthy 2012 [131]	50	Mixed	Y	Y	Y	N									Y	Y	Y	Y	Y	N	N
McCrindle 2011 [132]	50	Mixed	Y	Y	Y	N									Y	Y	Y	Y	Y	N	N
Timmerman 2016 [133]	50	Mixed	Y	Y	Y	N									Y	Y	Y	Y	Y	N	N
Willems 2015 [134]	50	Qual	Y	N	Y	N															
Ashurst 2014 [135]	25	Mixed	N	N	N	N									Y	Y	Y	N	Y	N	N
Badr 2016 [136]	25	Mixed	Y	Y	N	N									N	N	N	N	N	N	N
Buman 2016 [137]	25	Mixed	N	N	Y	N									Y	Y	Y	Y	N	N	N
Hallberg 2014 [138]	25	Qual	Y	N	N	N															
Hong 2013 [139]	25	Mixed	Y	Y	Y	N									Y	N	N	N	Y	N	N
Jongstra 2016 [140]	25	Qual	Y	N	N	N															
Shorten 2015 [141]	25	Qual	N	Y	N	N															
Thompson 2013 [142,143]	25	Mixed	Y	Y	Y	N	N	N	N	N									Y	N	N
