# Supplementary material for: Participatory Methods to Engage Health Service Users in the Development of Electronic Health Resources: Systematic Review
Source: J Particip Med. 2019 Feb 22;11(1):e11474. doi: 10.2196/11474 (PMC7434099; doi:10.2196/11474)
Supplement: Multimedia Appendix 3 [file jopm_v11i1e11474_app3.pdf]

Descriptive summary of 90 MMAT rated studies								
Study / References	Health condition or focus - MeSH	Purpose / Additional themes - MeSH	Technology Type	Age group: Youth 12-24; Adult 25-64; Aged 65+	Gender specific	LGBTQI+ specific	Cultural aspect	Country where studied
Ahtinen 2013 [26]	Health Promotion;	Exercise; Health Behavior; Healthy Lifestyle; Motivation;	Mobile Application	adult (25-55)	no	no	Multicultural - Aims to be applicable across cultures, but localized versions developed for Finland & India with content the same but nuances differing eg avatars. Countries chosen for variation in culture & environment.	Finland; India
Antypas 2014 [27]	Cardiovascular Diseases;	Cardiac Rehabilitation; Exercise; Health Behavior	Website; Mobile Application	adult,aged	no	no	no	Norway
Bengtsson 2014 [28, 29]	Cardiovascular Diseases; Hypertension;	Self Care;	Mobile Application	adult, aged (30+)	no	no	No - Not defined as specific to Sweden but inclusion criteria for participants was fluency in Swedish & only 2 participants were not from Sweden - noted as limitation	Sweden
Buccieri 2015 [30]	Health Promotion;	Homeless Persons; Health Services Accessibility	Mobile Application	youth	no	no	no	Canada
Clayman 2008 [31]	Neoplasms; Breast Neoplasms;	Patient Education as Topic;	Website	adult, aged	Female only	no	no	United States
Cordova 2015 [32]	Virus Diseases; Sexually Transmitted Diseases; HIV; Substance-Related Disorders;	Health Promotion; Power (Psychology); Primary Health Care;	Mobile Application	youth (12-21)	no	no	no - but aims to be tailored to each community	United States
Dabbs 2009 [33]	Respiratory Tract Diseases; Lung Transplantation;	Health Records, Personal;	Handheld Computer	youth, adult, aged (18+)	no	no	no	United States
Das 2013 [34]	Nutritional and Metabolic Diseases;	Food and Nutrition; Weight Reduction Programs; Follow-Up Studies;	Website	youth (18+); adult	no	no	no	Norway
Davies 2015 [35, 36]	Virus Diseases; Hepatitis B;	Patient Education as Topic; Health Promotion;	Mobile Application	adult	no	no	Cultural - Indigenous Australians. Bilingual - English and Yolnu Matha (language used in East Arnhem Land)	Australia
Fennell 2016 [39, 44]	Neoplasms;	Psychosocial Support Systems; Health Services Accessibility	Website	youth, adult, aged	no	no	no	Australia
Fonda 2010 [40, 41]	Endocrine System Diseases; Diabetes Mellitus;	Self Care; Patient Education as Topic; Health Records, Personal;	Mobile Application; Personal Health Record;	adult, aged	no	no	no	United States

Descriptive summary of 90 MMAT rated studies								
Study / References	Health condition or focus - MeSH	Purpose / Additional themes - MeSH	Technology Type	Age group: Youth 12-24; Adult 25-64; Aged 65+	Gender specific	LGBTQI+ specific	Cultural aspect	Country where studied
Goldenberg 2015 [42, 43]	Virus Diseases; Sexually Transmitted Diseases; HIV;	Patient Education as Topic;	Mobile Application	youth; adult (18+)	Male only	yes (men who have sex with men)	no	United States
Heckman 2015 [45]	Neoplasms; Skin Neoplasms;	Patient Education as Topic; Health Behavior;	Website	youth (20-25 years)	no	no	no	United States
Kelders 2013 [46]	Mental Disorders; Depressive Disorder;	Behavior Therapy; "Acceptance and Commitment Therapy"; Patient Education as Topic;	Website	adult	no	no	no	Netherlands
Lubberding 2015 [37, 38, 47]	Neoplasms;	Survivors; "Quality of Life"; Self Care; Healthy Lifestyle; Psychosocial Support Systems;	Website	adult, aged	no	no	no	Netherlands
Meyer 2007 [48]	Mental Disorders; Depressive Disorder;	Power (Psychology); Self Care; "Patient Acceptance of Health Care"	Website	youth (university students)	no	no	no	United Kingdom
Miller 2015 [49]	Neoplasms; Prostatic Neoplasms;	Survivors; Self Care; Adaptation, Psychological	Website	adult, aged	Male only	no	no	United States
Morrison 2015 [50]	Respiratory Tract Diseases; Asthma;	Self Care;	Website	youth; adult	no	no	no	United Kingdom
O'Brien 2016 [51]	Geriatrics;	Retirement; Patient Education as Topic;	Website	adult, aged	no	no	no	United Kingdom
Peute 2015 [52]	Neoplasms;	Survivors; Patient Education as Topic;	Website	youth	no	no	no	Netherlands
Revenas 2015 [53-55]	Musculoskeletal Diseases; Rheumatoid Diseases; Arthritis, Rheumatoid;	Exercise; Self Care;	Mobile Application	youth, adult, aged	no	no	no	Sweden
Sandlund 2015 [56]	Geriatrics;	Accidental Falls; Exercise; Motivation;	Mobile Application	aged	no	no	no	Sweden
Schnall 2014 [57]	Virus Diseases; Sexually Transmitted Diseases; HIV;	Patient Education as Topic; Health Behavior;	Mobile Application	youth, adult (13-64)	men only	yes - men who have sex with men (MSM)	No	United States

Descriptive summary of 90 MMAT rated studies								
Study / References	Health condition or focus - MeSH	Purpose / Additional themes - MeSH	Technology Type	Age group: Youth 12-24; Adult 25-64; Aged 65+	Gender specific	LGBTQI+ specific	Cultural aspect	Country where studied
Skjoth 2015 [58]	Women's Health;	Patient Education as Topic; Decision Making; Decision Support Techniques; Down Syndrome; Prenatal Diagnosis;	Website; Decision Tool	youth, adult	female only	no	no	Denmark
Stinson 2014 [59]	Nervous System Diseases; Pain; Chronic Pain	Self Care; Psychosocial Support Systems; Adaptation, Psychological; Patient Education as Topic;	Mobile Application	youth	no	no	no	Canada
van Bruinessen 2014 [60, 61]	Neoplasms; Lymphoma;	Communication; Physician-Patient Relations; Patient Education as Topic;	Website	youth, adult	no	no	no	Netherlands
Widman 2016 [62]	Virus Diseases; Sexually Transmitted Diseases; HIV;	Sex Education; Communication; Sexual Behavior; Health Behavior;	Website	youth (15-24)	Female only	no	No - specifically chose ethnically diverse participants	United States
Winterling 2016 [63-66]	Neoplasms;	Sex Education; Reproductive Health; Self Care; Patient Education as Topic;	Website	youth	no	no	no	Sweden
Ennis 2014 [67, 69]	Mental Disorders;	Health Records, Personal;	Personal Health Record	youth; adult	no	no	no	United Kingdom
Fleisher 2014 [68]	Neoplasms;	Patient Education as Topic; Decision Making; Decision Support Techniques;	Website; Decision Tool	adult, aged	no	no	no	United States
Alnasser 2016 [70,71]	Nutritional and Metabolic Diseases;	Food and Nutrition; Weight Reduction Programs;	Mobile Application	youth, adult (18+)	female only	no	Cultural - Arabic specific	Saudi Arabia
Alvarado-Martel 2015 [72]	Endocrine System Diseases; Diabetes, Mellitus;		Website	youth, adult (18+)	no	no	No	Spain
Andersen 2011 [74]	Cardiovascular Diseases; Heart Diseases;		Personal Health Record; Website; Telemonitoring	adult	no	no	no	Denmark
Armstrong 2007 [75-77]	Endocrine System Diseases; Diabetes Mellitus;		Website	youth, adult (16+)	no	no	no	United Kingdom
Atkinson 2009 [78]	Nutritional and Metabolic Diseases;	Food and Nutrition;	Website	youth, adult (18+)	female	no	no	United States

Descriptive summary of 90 MMAT rated studies								
Study / References	Health condition or focus - MeSH	Purpose / Additional themes - MeSH	Technology Type	Age group: Youth 12-24; Adult 25-64; Aged 65+	Gender specific	LGBTQI+ specific	Cultural aspect	Country where studied
Bae 2009 [79]	Mental Disorders; Depressive Disorder;		Website	adult	no	no	Yes - Korean specific	Republic of Korea
Boyd 2015 [81]	Cardiovascular Diseases;	Medication Therapy Management; Patient Education as Topic;	Mobile Application	aged	no	no	no	United States
Buckingham 2015 [82]	Mental Disorders;		Mobile Application	adult	no	no	no	United Kingdom
Cade 2013 [83,85]	Nutritional and Metabolic Diseases;	Food and Nutrition;	Website	youth, adults, aged	no	no	no	United Kingdom
Cafazzo 2012 [84]	Endocrine System Diseases; Diabetes Mellitus		Mobile Application	youth	no	no	no	Canada
Cnossen 2015 [86]	Respiratory Tract Diseases; Laryngectomy;		Website	adult, aged	no	no	no	Netherlands
Colombo 2016 [87]	Nervous System Diseases; Multiple Sclerosis;		Website	adult, aged	no	no	Multicultural -Italian & Australian project - this paper reports on the Italian project's website	Australia; Italy
Coyne 2016 [88]	Chronic Disease;		Website	youth	no	no	no	Ireland
Danaher 2012 [89]	Mental Disorders; Depressive Disorder;		Website	youth, adult (18+)	female only	no	no	Australia; United States;
Davies 2009 [90,123]	Mental Disorders; Dementia;	Geriatrics;	Wearable Device	aged	no	no	Multicultural - Trial sites across 3 countries	Netherlands; Sweden; Northern Ireland/UK
Dykes 2014 [91]	Neoplasms;		Website	youth (18+); adult; aged	no	no	no	United States
Enah 2014 [92,93]	Virus Diseases; Sexually Transmitted Diseases; HIV;	Sex Education;	Website; Serious Game	youth;	no	no	Cultural - African American	United States
Fink 2015 [94]	Geriatrics;		Website	aged	no	no	no	United States
Fledderus 2015 [95]	Nervous System Diseases; Pain; Chronic Pain		Website; Mobile Application	adult	no	no	no	Netherlands
Graham 2014 [96]	Women's Health; Female Urogenital Diseases and Pregnancy Complications;	Weight Reduction Programs; Female Urogenital Diseases and Pregnancy Complications;	Website	youth (18+); adult	female only	no	no	United States

Descriptive summary of 90 MMAT rated studies								
Study / References	Health condition or focus - MeSH	Purpose / Additional themes - MeSH	Technology Type	Age group: Youth 12-24; Adult 25-64; Aged 65+	Gender specific	LGBTQI+ specific	Cultural aspect	Country where studied
Hearn 2014 [97,98]	Women's Health; Female Urogenital Diseases and Pregnancy Complications;		Website; Mobile Application	adult	female only	no	no	Australia
Hightow-Weidman 2011 [99]	Virus Diseases; Sexually Transmitted Diseases; HIV;	Sex Education;	Website	youth (18+); adult	male only	yes; MSM	Cultural - African American	United States
Horne 2016 [100]	Neoplasms;		Website	adult	no	no	no	United Kingdom
Kattelmann 2014 [101]	Nutritional and Metabolic Diseases;	Food and Nutrition;; Weight Reduction Programs; Healthy Lifestyle	Website	youth (18-24);	no	no	no	United States
Kim 2015 [102]	Nutritional and Metabolic Diseases;	Food and Nutrition; Weight Reduction Programs;	Mobile Application	youth (13-24);	no	no	Cultural - low income, urban, minority youth	United States
Kuijpers 2015 [103,104]	Neoplasms;	Power (Psychology);	Website	adult;	no	no	no	Netherlands
Lee 2013 [105]	Neoplasms;	Food and Nutrition;	Website	adult	no	no	no	Republic of Korea
Martin-Hammond 2015 [106]	Geriatrics;	Medication Therapy Management;	Decision Tool; Kiosk Application;	aged	no	no	no	United States
Neville 2016 [107,108]	Immune System Diseases; Lupus Erythematosus, Systemic;		Website	adults	no	no	no	Canada
Schlosser 2016 [109]	Mental Disorders;		Mobile Application	youth (16+); adult	no	no	no	United States
Siek 2011 [110]	Geriatrics;	Medication Therapy Management;	Website	aged	no	no	no	United States
Todd 2013 [111,122]	Mental Disorders; Bipolar Disorder;		Website	adult	no	no	no	United Kingdom
Trudeau 2011 [112]	Women's Health; Menopause	Patient Education as Topic;	Website	adult	female only	no	no	United States
Vonk Noordegraaf 2012 [113]	Women's Health		Website	youth (18+); adult	female only	no	no	Netherlands
Ward 2016 [114]	Hemic and Lymphatic Diseases; Hematologic Diseases; Thalassemia		Mobile Application	youth (18+); adult	no	no	no	Canada; United States
Waterlander 2014 [115]	Nutritional and Metabolic Diseases;	Food and Nutrition; Weight Reduction Programs;	Mobile Application	youth (18+); adult	no	no	no	New Zealand
Whitehouse 2013 [116]	Mental Disorders;		Mobile Application	youth (12-18);	no	no	no	Canada

Descriptive summary of 90 MMAT rated studies								
Study / References	Health condition or focus - MeSH	Purpose / Additional themes - MeSH	Technology Type	Age group: Youth 12-24; Adult 25-64; Aged 65+	Gender specific	LGBTQI+ specific	Cultural aspect	Country where studied
Whittaker 2008 [117]	Substance-Related Disorders; Tobacco Use Disorder;		Mobile Application	youth	no	no	no	New Zealand
Williamson 2016 [80,118,119]	Skin and Connective Tissue Diseases;		Website	adult	no	no	no	United Kingdom
Wright 2016 [120]	Substance-Related Disorders; Alcohol-Related Disorders		Mobile Application	youth;	no	no	no	Australia
Wysocki 2016 [121]	Endocrine System Diseases; Diabetes Mellitus;		Website; Decision Tool	youth; adults (adolescents & parents)	no	no	no	United States
Crosby 2016 [124]	Hemic and Lymphatic Diseases; Hematologic Diseases; Anemia, Sickle Cell;		Mobile Application	youth (16-24);	no	no	Cultural - mostly African American	United States
Foster 2015 [125]	Neoplasms;		Website	adult	no	no	no	United Kingdom
Gordon 2015 [126]	Urologic Diseases; Kidney Diseases;		Website	adult	no	no	Cultural - Hispanic	United States
Grant 2012 [127]	Cardiovascular Diseases; Hypertension;	Medication Therapy Management;	Website	adult	no	no	no	United States
Groussard 2015 [128]	Nervous System Diseases; Trauma, Nervous System; Craniocerebral Trauma; Brain Injuries;		Mobile Application	adult	no	no	no	Canada
Hallett 2009 [129]	Substance-Related Disorders; Alcohol-Related Disorders		Website	youth (17-24);	no	no	no	New Zealand
Heinrich 2009 [130]	Endocrine System Diseases; Diabetes Mellitus;		Website	adult;	no	no	no	Netherlands
McCarthy 2012 [131]	Virus Diseases; Sexually Transmitted Diseases;	Sex Education;	Website	youth (16-22);	no	no	no	United Kingdom
McCrindle 2011 [132]	Geriatrics;		Wearable Device	aged	no	no	Multicultural - Studied across 6 European countries	Austria; Belgium; Czech Republic; Greece; Spain; United Kingdom;
Timmerman 2016 [133]	Neoplasms; Lung Neoplasms;		Mobile Application	adult; aged	no	no	no	Netherlands
Willems 2015 [134]	Neoplasms;	Survivors;	Website	youth (18+), adult	no	no	no	Netherlands

Descriptive summary of 90 MMAT rated studies								
Study / References	Health condition or focus - MeSH	Purpose / Additional themes - MeSH	Technology Type	Age group: Youth 12-24; Adult 25-64; Aged 65+	Gender specific	LGBTQI+ specific	Cultural aspect	Country where studied
Ashurst 2014 [135]	Endocrine System Diseases; Diabetes Mellitus;		Mobile Application	youth (16-25);	no	no	no	United Kingdom
Badr 2016 [136]	Neoplasms;	Survivors;	Website	adult	no	no	no	United States
Buman 2016 [137]	Nutritional and Metabolic Diseases;	Healthy Lifestyle	Mobile Application	adult	no	no	no	United States
Hallberg 2014 [138]	Cardiovascular Diseases; Hypertension;		Mobile Application	adult	no	no	no	Sweden
Hong 2013 [139]	Neoplasms;	Survivors;	Mobile Application	adult (60+); aged	no	no	no	United States
Jongstra 2016 [140]	Cardiovascular Diseases;		Website	elderly	no	no	Multicultural - Across 3 European countries	Finland; France; Netherlands;
Shorten 2015 [141]	Women's Health; Female Urogenital Diseases and Pregnancy Complications;		Decision Tool; Website	adult	female only	no	Cultural - Initial resource was designed for Australian women and adapted to meet needs of American women	United States
Thompson 2013 [142,143]	Nutritional and Metabolic Diseases;	Exercise; Health Behavior; Healthy Lifestyle; Motivation;	Website	youth (12-17);	no	no	no	United States
