# Supplementary material for: Participatory Methods to Engage Health Service Users in the Development of Electronic Health Resources: Systematic Review
Source: J Particip Med. 2019 Feb 22;11(1):e11474. doi: 10.2196/11474 (PMC7434099; doi:10.2196/11474)
Supplement: Multimedia Appendix 4 [file jopm_v11i1e11474_app4.pdf]

Major health focus [MeSH Terms]	Total MMAT rated studies n=90 (%)	References (some studies may include more than one reference)
Neoplasms (cancer)	18 (20%)	[31, 37-39, 44, 45, 47, 49, 52, 60, 61, 63-66, 68, 91, 100, 103-105, 125, 133, 134, 136, 139]
Mental Disorders	10 (11%)	[46, 48, 67, 69, 79, 82, 89, 90, 109, 111, 116, 122, 123]
Nutritional and Metabolic Diseases	9 (10%)	[34, 70, 71, 78, 83, 85, 101, 102, 115, 137, 142, 143]
Virus Diseases (eg HIV)	8 (9%)	[32, 35, 36, 42, 43, 57, 62, 92, 93, 99, 131]
Cardiovascular Diseases	7 (8%)	[27-29, 74, 81, 127, 138, 140]
Endocrine System Diseases (eg Diabetes)	7 (8%)	[40, 41, 72, 75-77, 84, 121, 130, 135]
Geriatrics	6 (7%)	[51, 56, 94, 106, 110, 132]
Women's Health	6 (7%)	[58, 96-98, 112, 113, 141]
Nervous System Diseases	4 (4%)	[59, 87, 95, 128]
Respiratory Tract Diseases	3 (3%)	[33, 50, 86]
Substance-Related Disorders	3 (3%)	[117, 120, 129]
Health Promotion	2 (2%)	[26, 30]
Hemic and Lymphatic Diseases	2 (2%)	[114, 124]
Chronic Disease	1 (1%)	[88]
Immune System Diseases	1 (1%)	[107, 108]
Musculoskeletal Diseases	1 (1%)	[53-55]
Skin and Connective Tissue Diseases	1 (1%)	[80, 118, 119]
Urologic Diseases	1 (1%)	[126]
