# Supplementary material for: Participatory Methods to Engage Health Service Users in the Development of Electronic Health Resources: Systematic Review
Source: J Particip Med. 2019 Feb 22;11(1):e11474. doi: 10.2196/11474 (PMC7434099; doi:10.2196/11474)
Supplement: Multimedia Appendix 5 [file jopm_v11i1e11474_app5.pdf]

Technology	Total MMAT rated studies n=90 (%)	References (studies may include more than one reference)
Website	56 (62%)	[27, 31, 34, 37-39, 44-52, 58, 60-66, 68, 72, 74-80, 83, 85-89, 91-101, 103-105, 107, 108, 110-113, 118, 119, 121, 122, 125-127, 129-131, 134, 136, 140-143]
Mobile Application	32 (36%)	[26-30, 32, 35, 36, 40-43, 53-57, 59, 70, 71, 81, 82, 84, 95, 97, 98, 102, 109, 114-117, 120, 124, 128, 133, 135, 137-139]
Decision Tool	5 (6%)	[58, 68, 106, 121, 141]
Personal Health Record	3 (3%)	[40, 41, 67, 69, 74]
Wearable Device	2 (2%)	[90, 123, 132]
Handheld Computer	1 (1%)	[33]
Kiosk Application	1 (1%)	[106]
Serious Game	1 (1%)	[92, 93]
Telemonitoring	1 (1%)	[74]
