# Supplementary material for: Participatory Methods to Engage Health Service Users in the Development of Electronic Health Resources: Systematic Review
Source: J Particip Med. 2019 Feb 22;11(1):e11474. doi: 10.2196/11474 (PMC7434099; doi:10.2196/11474)
Supplement: Multimedia Appendix 6 [file jopm_v11i1e11474_app6.pdf]

**Targeted population**	**Total MMAT rated studies n=90 (%)**	**References (studies may include more than one reference)**
Youth (12-24)	22 (24%)	[30, 32, 45, 48, 52, 59, 62-66, 84, 88, 92, 93, 101, 102, 116, 117, 120, 121, 124, 129, 131, 135, 142, 143]
Aged (65+)	9 (10%)	[56, 81, 90, 94, 106, 110, 123, 132, 139, 140]
Women	11 (12%)	[31, 58, 62, 70, 71, 78, 89, 96-98, 112, 113, 141]
Men	4 (4%)	[42, 43, 49, 57, 99]
LGBTQI+	3 (3%)	[42, 43, 57, 99]
Cultural /multicultural focus	14 (16%)	[26, 35, 36, 70, 71, 79, 87, 90, 92, 93, 99, 102, 123, 124, 126, 132, 140, 141]
