# Supplementary material for: Participatory Methods to Engage Health Service Users in the Development of Electronic Health Resources: Systematic Review
Source: J Particip Med. 2019 Feb 22;11(1):e11474. doi: 10.2196/11474 (PMC7434099; doi:10.2196/11474)
Supplement: Multimedia Appendix 7 [file jopm_v11i1e11474_app7.pdf]

Country of study	Total MMAT rated studies n=90 (%)	References (studies may include more than one reference)
USA	33 (37%)	[31-33, 40-43, 45, 49, 57, 62, 68, 78, 81, 91-94, 96, 99, 101, 102, 106, 109, 110, 112, 114, 121, 124, 126, 127, 136, 137, 139, 141-143]
UK	15 (17%)	[48, 50, 51, 67, 69, 75-77, 80, 82, 83, 85, 90, 100, 111, 118, 119, 122, 123, 125, 131, 132, 135]
Netherlands	13 (14%)	[37, 38, 46, 47, 52, 60, 61, 86, 90, 95, 103, 104, 113, 123, 130, 133, 134, 140]
Canada	7 (8%)	[30, 59, 84, 107, 108, 114, 116, 128]
Australia	6 (7%)	[35, 36, 39, 44, 87, 89, 97, 98, 120]
Sweden	6 (7%)	[28, 29, 53-56, 63-66, 90, 123, 138]
New Zealand	3 (3%)	[115, 117, 129]
Denmark	2 (2%)	[58, 74]
Finland	2 (2%)	[26, 140]
Spain	2 (2%)	[72, 132]
Norway	2 (2%)	[27, 34]
Republic of Korea	2 (2%)	[79, 105]
Austria	1 (1%)	[132]
Belgium	1 (1%)	[132]
Czech Republic	1 (1%)	[132]
France	1 (1%)	[140]
Greece	1 (1%)	[132]
India	1 (1%)	[26]
Ireland	1 (1%)	[88]
Italy	1 (1%)	[87]
Saudi Arabia	1 (1%)	[70, 71]
