# Supplementary material for: Participatory Methods to Engage Health Service Users in the Development of Electronic Health Resources: Systematic Review
Source: J Particip Med. 2019 Feb 22;11(1):e11474. doi: 10.2196/11474 (PMC7434099; doi:10.2196/11474)
Supplement: Multimedia Appendix 8 [file jopm_v11i1e11474_app8.pdf]

Detail of data extracted from 30 studies scoring ≥ 90% on MMAT (additional information may have been reported in literature that was not reviewed)										
Study / References	Specific product	Frameworks, models, theories	Health service user (HSU) population	Health Service Provider (HSP) population	Teams or groups	CeHRes roadmap: Contextual inquiry	CeHRes roadmap:Value Specification	CeHRes roadmap: Design	CeHRes roadmap: Operationalization	CeHRes roadmap: Summative Evaluation
Ahtinen 2013 [26]	"Living Application" - mobile wellness application to support physical activity	Transtheoretical Model (Prochaska Stages of Change); Constructive Design Research; Social Cognitive Theory; Persuasive System Design model (PSD)	urban; sedentary work; interested in wellness management; studies across both Finland and India; age range 24-70;	none defined	No named team, but mention of multicultural group of researchers & designers	1st field study with 16 HSU from 2 countries - process included explorative study with wellness diary including: pre study semi structured face to face interviews; 2 week use of wellness diary app, phone interviews during trial and post trial interview (HSU n=16); audio recordings; thematic analysis;	2nd field study with 12 HSU in 2 countries - participatory design study with a Magical Gadget (imaginative mock up SPES method) including pre study phone interviews, participatory design task, think aloud; observation and post study interview;	design principles derived using affinity wall technique; iterative development in workshops (IT experts & user experience practitioners);	Not reported	Not reported
Antypas 2014 [27]	website & app connected to Skibotn Rehabilitation Center. Includes personal profile, activity calendar, SMS reminders	Transtheoretical Model (Prochaska Stages of Change); Health Behaviour Theory; Social Cognitive Theory; computer tailoring; decisional balance; Health Action Process Approach (HAPA); Regulatory Focus Theory (RFT)	men & women participating in cardiac rehabilitation program at the Skibotn Rehabilitation Centre in Norway; women mean age 64.3 & men mean age 59.4;	Other than 2 researchers, HSP involved with the project by developing & posting information on the website are not defined	Researchers: cardiovascular nurse & health psychologist	focus group based on interview guide with open discussion (HSU n=11); audio recording; thematic analysis;	audio recording; thematic analysis;	development of personas & narratives; 2 localized versions of concept scenario developed for Finland & India;	Not reported	randomized controlled trial - clinicaltrials.gov NCT01223170
Bengtsson 2014 [28, 29]	mobile phone self report system for self management of hypertension	participatory research design	men and women aged 49-82; currently using hypertension drugs, fluent in Swedish; recruitment for evaluation from 2 geographically separate locations - primary healthcare centre in multicultural city suburb & outpatient clinic of	HSPs recruited from same units as HSUs; included equal numbers of physicians,nurses & pharmacists; experienced in caring for patients with hypertension	unnamed interdisciplinary group of researchers;	Ethics approval; 3 x patient semi structured focus groups (HSU n=15); 2 x clinician semi structured focus groups (HSP n=12); audio recording; thematic analysis using NVivo;	see focus group in "contextual inquiry"	brief evaluation sessions of initial concept model on paper (participants not defined); prototype; 4 x semi structured focus groups evaluating Living Aplication concept (HSU in 2 countries n=19); audio & video recording; thematic analysis by multicultural group of researchers & designers"	Not reported	Not reported

Detail of data extracted from 30 studies scoring ≥ 90% on MMAT (additional information may have been reported in literature that was not reviewed)										
Study / References	Specific product	Frameworks, models, theories	Health service user (HSU) population	Health Service Provider (HSP) population	Teams or groups	CeHRes roadmap: Contextual inquiry	CeHRes roadmap:Value Specification	CeHRes roadmap: Design	CeHRes roadmap: Operationalization	CeHRes roadmap: Summative Evaluation
			a provincial hospital in smaller town							
Buccieri 2015 [30]	Supporting and Assisting Youth (SAY) mobile app for homeless youth	Spiral Technology Action Research (STAR) model; critical social theory of youth empowerment; action research; youth centered participatory action study	12 homeless youth; 18-23 years;	2 university facilitators	SAY App Committee: youth (HSU n=12) and university facilitators (n=2)	literature review; first of series of group discussions for "Listen" stage (HSU n=12, researchers n=2) (Note: 10 group discussions in total across all development stages)	item drafting based on focus group results; literature review;	development of functional prototype over 1 year using combination of theoretical frameworks and focus group results (researchers & developers);	SAY App Committeee worked on promotional campaign including design of poster mailed to 250 youth focused social service agencies across Canada, & promotion via Twitter & Facebook (HSU n=12, researchers n=2); SAY app available for free download from 2013	Not reported
Clayman 2008 [31]	Cancer Care Links (www.cancercarelinks.org) - website for women diagnosed with breast cancer	Not reported	women diagnosed with breast cancer; recruited at National Cancer Institute designated Comprehensive Cancer Centre; aged 29-80	surgical oncologists, medical oncologists, radiation oncologists, plastic surgeons, nurses & therapists; recruited at National Cancer Institute designated Comprehensive Cancer Centre	Core research team/research & development team: faculty with expertise in communication science, cancer communicatrion, public health, clinical psychology, health education & multimedia production (number not specified) plus 1 breast cancer survivor as research assistant and 1 multimedia specialist	longitudinal series of semi structured interviews re information needs & resource use including after 1st appointment with oncologist & follow up telephone interviews fortnightly for 6 months (HSU n=30); one-time provider interviews (HSP n=22) ; audio recording; thematic analysis;	further group discussions for "Plan" stage (HSU n=12, researchers n=2) (Note 10 group discussions in total across all development stages)	several design meetings (participants not defined); iterative testing for content validity & usability evaluation by 4 x face to face cognitive interviews (HSU n=21, HSP n=4); audio recording; Item Tracking Matrix & Cognitive Interview Summary used for data analysis;	Not reported	Not reported

Detail of data extracted from 30 studies scoring ≥ 90% on MMAT (additional information may have been reported in literature that was not reviewed)										
Study / References	Specific product	Frameworks, models, theories	Health service user (HSU) population	Health Service Provider (HSP) population	Teams or groups	CeHRes roadmap: Contextual inquiry	CeHRes roadmap:Value Specification	CeHRes roadmap: Design	CeHRes roadmap: Operationalization	CeHRes roadmap: Summative Evaluation
Cordova 2015 [32]	mobile app version of Storytelling for Empowerment (S4E) - HIV/STI and drug abuse preventive intervention for primary care	community-based participatory research (CBPR); prevention principles of the National Institute on Drug Abuse (NIDA); eco developmental framework; agile software development	29 adolescents recruited at youth centered primary care clinic; mostly African American (65%) and female (72%); urban; age range 12-18	not specified	Youth Leadership Council (YLC): diverse youth led group advocating for change & partnering with universities (no detail on participants);	Involvement of YLC in all aspects of study including proposal submission, development of interview guide, participant recruitment, content identification, manuscript preparation; 9 x focus group interviews using iterative interview guide (HSU n=25); individual interviews (HSU n=4); audio recording; thematic analysis;	Combined with contextual inquiry processes - see previous;	remainder of series of 10 x group discussions for "Do" stage; 10 x team meetings (HSU n=12, researchers n=2) including planning sketching ideas, making concrete decisions, formatting the app, developing paper prototype; testing of electronic prototype by group (HSU n=12);	Not reported	Not reported
Dabbs 2009 [33]	Pocket Personal Assistant for Tracking Health (Pocket PATH) - for lung transplant patients	user centred design	7 lung transplant patients; aged 21-69; recruited from pulmonary transplant center; 12+ months since transplant; English speaking	See Development Team;		Data collection re background characteristics of patients impacting on computer use from various sources eg previous client survey, symptom review; literature review; field interview (home visit) and observation including contextual inquiry methods (HSU n=7);	Combined with contextual inquiry processes - see previous;	development team meetings - iterative development in response to results of ongoing interviews; continued semi structured interviews for ongoing feedback, testing of prototypes (HSU n=30); beta test including anonymous questionnaire (HSU n=12)	Not reported	testing of final version (HSU n=6); Post-Study System Usability Questionnaire (PSSUQ); Randomized controlled pilot trial found newly transplanted patients found Pocket PATH superior to conventional methjods for self monitoring after transplantation. full RCT; ongoing evaluation involving end users after RCTs to inform continued development;

Detail of data extracted from 30 studies scoring ≥ 90% on MMAT (additional information may have been reported in literature that was not reviewed)										
Study / References	Specific product	Frameworks, models, theories	Health service user (HSU) population	Health Service Provider (HSP) population	Teams or groups	CeHRes roadmap: Contextual inquiry	CeHRes roadmap:Value Specification	CeHRes roadmap: Design	CeHRes roadmap: Operationalization	CeHRes roadmap: Summative Evaluation
Das 2013 [34]	ehealth portal for weight loss patients undergoing treatment	human centred design as per ISO 9241-210 (2008) ; iterative design approach (includes diagram); participatory design;	Norwegian speakers; completed hospital weight reduction program - either lifestyle therapy or bariatric surgery; age range 31-55;	8 health professionals with experience in field of obesity from fields of nursing (n=6); medicine (n=1) & clinical nutrition (n=1); aged 26-64;	Research team: lead author + trained research assistants"	Ethics approval; field study including 10 x observations of group & individual patient consultations with staff and semi structured interviews (HSP n=not reported) over 1 month; thematic analysis;	combined with contextual inquiry processes - see previous;	iterative app programming synergistic with HSU interviews (agile software development); prototypes shared with YLC & HSU; weekly meetings with programmer;	implementation at the obesity clinic at the hospital for case study.	Not reported
Davies 2015 [35, 36]	"Hep B Story" - a culturally appropriate bilingual mobile app about hepatitis B for indigenous Australians.	participatory action research (PAR) framework; Paasche-Orlow & Wolf's model used as organising model for data analysis - "The causal pathways linking health literacy to health outcomes";	indigenous patients with Hep B; recruited at health clinic of remote community in Arnhem Land; age range 30-59;	See Project Team	Development Team: interdisciplinary - included nurse (principle investigator), computer scientists, behavioral scientists, cardiothoracic transplant physician, communication scientist	Ethics approval; Published qualitative study including 32 x semistructured interviews with indigenous patients with hep B (HSU n=11); indigenous community members (n=9), key informants (n=12, 4 of whom were indigenous), in total 24 of the group were indigenous; thematic analysis based on Paasche-Olow & Wolf model;	3 x participatory design workshops: 1st with HSP (n=8) ; 2nd with HSU who had lifestyle therapy (HSU n=6); 3rd with HSU who had bariatric surgery (HSU n=6); workshops included semi structured group interviews, design activities, sketching ideas, group presentation of ideas, personas, scenarios; workshop evaluation; audio & video recording & transcription; thematic analysis;	3 x User centered design (UCD) sessions in laboratory setting using low fidelity prototypes, observation and screen capture using Camtasia (HSU n=7); usability assessment reports generated for all testing sessions & reviewed by development team; iterative cycles of assessing-designing-testing-analyzing-refining-testing-analyzing-refining; field study testing Pocket PATH at home (HSU n=6);	community launch event including presentation of app & invitation for initial evaluation; launched at inaugural Indigenous People's Conference on Viral Hepatitis;	initial evaluation questionnaire including sections pre & post exploring the app (n=16 community end users at community launch event); evaluation questionnaire with conference delegates (n-56)
Fennell 2016 [39, 44]	Country Cancer Support website	participatory action research (PAR) framework informed by Hart & Bond's 1995 framework; transtheoretical model; behaviour change theories including Prochaska Stages of Change & Health Belief Model;	PAR Partners & also website visitors over 18 with diagnosis of cancer or carer/relative/friend of someone diagnosed with cancer living in rural area; aged 18-74;	Professional Reviewers: Oncologist, Helpline Nurse Counsellors, Psychologists)	Not reported	Ethics approval; formative research - published qualitative study identified need for resource including semi structured face to face interviews (HSU n=17); demographic questionnaire (HSU n=17); thematic analysis; review of other websites; review of clinical practice guidelines; literature review; funding consultations with government & non government stakeholders;	[Combined with contextual inquiry processes - see previous];	development of first prototype; evaluation workshop for 1st prototype (all stakeholder groups inc HSU n=6); workshop included researcher presentations, stakeholder feedback, plenary discussions, break-out groups, CARD-sorting (Collaborative Analysis of Requirements and Design); audio & video recording; development of 2nd prototype; usability tests in	launched with PAR partners as ambassadors (HSU n=11) & rural media; promotional tour;	Google analytics results from first 3 years for quantitative metrics, analysed using Statistical Package for the Social Sciences (SPSS); acceptability testing web survey with adaptive questioning for website visitors (HSU=108);

Detail of data extracted from 30 studies scoring ≥ 90% on MMAT (additional information may have been reported in literature that was not reviewed)										
Study / References	Specific product	Frameworks, models, theories	Health service user (HSU) population	Health Service Provider (HSP) population	Teams or groups	CeHRes roadmap: Contextual inquiry	CeHRes roadmap:Value Specification	CeHRes roadmap: Design	CeHRes roadmap: Operational-ization	CeHRes roadmap: Summative Evaluation
								laboratory on 2nd prototype including questionnaire and semi structured post-test interview, audio & video recording, screen capture during test, think aloud & System Usability Scale (SUS) (n=20 including HSU n= 6); redesign after usability tests;		qualitative content analysis
Fonda 2010 [40, 41]	My Diabetes Data Tracker gadget - prototype of Personal health application for diabetes self management;	user centred design (UCD); Project Health Design (PHD)	adults with diabetes from Joslin Diabetes Center (Boston, MA); average age 63;	Not defined	Project Team: publication authors including reps from Charles Darwin University, Miwatj Aboriginal Health Corporation, Royal Darwin Hospital Liver Clinic, Dreamedia (n=6)	developed facilitators guide for focus group with person with diabetes (HSU n=1); 3 x focus groups to ascertain needs (HSU n=21); audio recording; thematic analysis;	Combined with contextual inquiry processes - see previous;	storyboard developed by Project team; 20+ iterative modifications to storyboard with community review; translation both forwards & backwards; 4 x functional prototypes developed iteratively with community review (HSU + HSP);	Not yet operational at date of publication	Funded for a 6 month prospective randomized trial of a mobile version of the PHA but not yet reported
Goldenberg 2015 [42, 43]	mobile HIV prevention app for men who have sex with men	iterative reseach process; iterative qualitative approach	Men who have sex with men from Atlanta, Seattle & rural US regions; aged 18+; owned or had owned a smartphone; never had positive HIV test	HIV testing counsellors in Atlanta and Seattle; aged 18+; owned or had owned a smartphone	PAR Partners: 4 men & 7 women living in "accessible" to "very remote" rural areas of South Australia, including a young person and a rural health care professional who had cared for her rural cancer affected father (represented HSPs and carers) ;	Ethics approval; 4 x in person focus groups with MSM (HSU n=28); 1 x online focus group with rural MSM (HSU n=10); 2 x in person focus groups with HIV testing counselors (HSP n=13); 14 key informant interviews; audio recording; thematic analysis;	Combined with contextual inquiry processes - see previous;	draft website plan based on initial study; demographic questionnaire (HSU n= 11); iterative development of website plan after each of 11 x individual telephone interviews (HSU n=11) - participatory action research cycle with PAR partners (HSU n=11); expert review of content by HSP; incorporated behavioural change strategies; consultation with HSU re online prototype (n=?) ; iterative development as result of review & consultation;	Not reported	Not reported

Detail of data extracted from 30 studies scoring ≥ 90% on MMAT (additional information may have been reported in literature that was not reviewed)

Study / References	Specific product	Frameworks, models, theories	Health service user (HSU) population	Health Service Provider (HSP) population	Teams or groups	CeHRes roadmap: Contextual inquiry	CeHRes roadmap:Value Specification	CeHRes roadmap: Design	CeHRes roadmap: Operationalization	CeHRes roadmap: Summative Evaluation
Heckman 2015 [45]	online skin cancer risk reduction intervention for young adults - UV4.me	Integrative Model of Behavior Prediction	25 young adults participating in an in-person skin cancer prevention randomized controlled intervention trial from a university campus in Phildelphia; aged 20-25; 64% white, 72% female		Project T eam: not defined	individual interviews including think aloud re baseline survey and pamphlet from prior intervention (HSU n=25); funding secured;	[combined with contextual inquiry processes - see previous];	prototype design and evaluation; 2 x focus groups re prototype ideas (HSU n=21); step by step demonstration of prototype to HSU to obtain feedback; group meeting to review prototype (HSP n=10); Planned & funded for next stages but not reported;	Not reported	pilot testing by 18-25 year olds at risk of developing skin cancer (HSU n=53); website refined; Randomized Controlled Trial in 2014 to compare UV4.me to existing Skin Cancer Foundation website & assessment only condition
Kelders 2013 [46]	web based intervention for prevention of depression, based on self help book "Living to the full"	CeHRes roadmap for eHealth development (figure included); human centered design; persuasive technology theory; ; business modeling	people between 26-62 years interested in participating in previous study of effectiveness of "Living to the full" (initial interviews); participants part of target group for intervention recruited with online advertisements (for usability testing)	ehealth researchers/usability experts from University of Twente including 1 clinical psychologist	Key Informants: primary care providers + key stakeholders at community based organisations, health departments & government agencies at local, county, state & federal levels;	literature scan; discussions re goals and needs with project management team);	2x focus groups (HSU n=4) to shape content, review potential images & suggest topics	beta version (prototype) designed; 6 x focus groups to theatre test beta version of app with MSM (HSU n=34); 2 x in person theatre testing focus groups (HSP n=9); audio recording; thematic analysis;	designed as research tool - this aspect not discussed	Not reported
Lubberding 2015 [37, 38, 47]	OncoKompas -- https://www.oncokompas.nl/ online self management application for cancer survivors	participatory design principles (van Gemert-Pijnen et al 2011, van der Vaart et al 2012)	mix of head & neck/breast cancer survivors chosen because different needs and gender spread; most female 67%; mean time since treatment 13.5 months; outpatients of	health care profesionals involved in head and neck cancer care, including surgeons, radiation oncologist, oncology nurses, health scientist, psychologist, spiritual	Multidisciplinary Team: Individuals with expertise in skin cancer prevention, young adults, internet interventions, psychology, qualitative & quantitative research methods, health literacy, computer	Ethics approval; qualitative needs assessment via semi structured interviews with HSU (n=30); 1st round of interviews with HSP re current follow up care (HSP n=11); audio recording; thematic analysis;	18 x semi structured interviews combined with rapid prototyping using paper prototypes with potential end users (HSU n=18); audio recording; thematic analysis of interviews & rapid prototyping; requirement session with project management team using results from thematic	preliminary content / mock up drafted; 4 x focus groups to review mock ups/prototype & provide suggestions for website (HSU n=10); thematic analysis; design development by Multidisciplinary Team; avatar development with feedback from HSU (n=26); health literacy evaluation	Discussions re implementation included in previous interviews with HSPs - see design processes; Feasibility study in clinical practice - pre-test survey before access to OncoKompas (HSU n=56) ; post	See feasibility study

Detail of data extracted from 30 studies scoring ≥ 90% on MMAT (additional information may have been reported in literature that was not reviewed)										
Study / References	Specific product	Frameworks, models, theories	Health service user (HSU) population	Health Service Provider (HSP) population	Teams or groups	CeHRes roadmap: Contextual inquiry	CeHRes roadmap:Value Specification	CeHRes roadmap: Design	CeHRes roadmap: Operationalization	CeHRes roadmap: Summative Evaluation
			VU University Medical Centre in Amsterdam; Dutch speaking; age range 48-88;	counsellor, patient advisor	programming, psychometrics and instructional design;		analysis;	by experts including readability evaluation using Precise SMOG ("Simple Measure of Gobbledygook") (experts n=2); pre-test questionnaire using cognitive interviewing with young adults including think aloud (HSU n-20); 2 rounds of acceptability testing using structured interviews (HSU n-26); usability testing including structured interviews (HSU n=12); quality control testing (project manager & programmers);	test survey 2 weeks after access (HSU n=56); follow up interview (HSU n=56); data analysis	
Meyer 2007 [48]	www.studentdepression.org - student focused website for depression self help	action research framework; action research spiral; social constructionist philosophy;	university students; youth (age of students participating not provided)	Author (principal project developer) is Dpsych candidate; expert validation panel of psychologists & a psychiatrist	"Project team (not defined);	project application formulated & submitted to charity; ethical approval; recruitment of research assistant to conduct interviews; recruitment of 'student consultants' advertised nationwide through UK universities; 13 x semi structured interviews with HSU (n=13);	2nd round of interviews re added value for HSP of ehealth tool (HSP n=11); audio recording; thematic analysis;	working prototype; user based usability evaluation using scenario based think aloud protocol on a working prototype (HSU n-10); audio & video recording; data analysis using coding; expert based usability inspection using cognitive walkthrough (HSP n=8); requirement session with program development team;	approval by steering group and charity board; launch	usage statistics - 50,000+ visits in first year, 100,000+ in second year; anonymous site feedback form with ratings
Miller 2015 [49]	PROGRESS: web based education program for prostate cancer survivors transitioning from active treatment; Prostate Cancer Online Guide & Resource for Electronic Survivorship (PRO-	behavioral science theory; Cognitive-Social Health Information Processing model (C-SHIP); iterative design; evidence based behavioral science theory; health communication best	early stage prostate cancer patients; aged 52-79 [Note that later in the paper these are referred to as "survivors")	clinicians from variety of specialties eg male reproductive medicine, psychology		literature review; input from interdisciplinary expert team re needs; review and identification of resources for content;	rough draft outline of website content; questionnaire for initial feedback on draft outline (HSU n=10); anonymous email discussion group to debate every aspect of website design (HSU n=10); reflective note taking & written summaries at key points (researcher); interview transcripts edited to form	discussion with Development Team re results of both needs assessments; prototype by Development Team; "demo sessions" re prototype and refinements; contents developed with experts including HSU, HSP and paramedics (no additional details); 11 x cognitive walkthroughs in pairs including expert	not reported	RCT of PROGRESS (website vs NCI's Facing Forward materials)

Detail of data extracted from 30 studies scoring ≥ 90% on MMAT (additional information may have been reported in literature that was not reviewed)										
Study / References	Specific product	Frameworks, models, theories	Health service user (HSU) population	Health Service Provider (HSP) population	Teams or groups	CeHRes roadmap: Contextual inquiry	CeHRes roadmap:Value Specification	CeHRes roadmap: Design	CeHRes roadmap: Operationalization	CeHRes roadmap: Summative Evaluation
	GRESS)	practices;					student approved "student stories" for content (HSU n=10);	usability evaluation and think aloud followed by semi structured interviews (HSP n=22); recording using Morae software; thematic analysis;		
Morrison 2015 [50]	"Living well with Asthma" - online resource for self management of asthma	iterative user centred design; implementation and behaviour change theories; Medical Research Council guide to developing and evaluating complex interventions (MRC) https://www.mrc.ac.uk/documents/pdf/complex-interventions-guidance/	adults with asthma; age range 23-56 (n=10)	practice nurses who undertake asthma reviews (n=4)	Project management team: researchers, designers & programmers (not specifically defined)"	Ethics approval; literature review; identification of features required by expert panel (experts n=7); implementation theory Normalization Process Theory (NPT) to inform topic guide for focus groups; 2 x focus groups re barriers & facilitators of self management interventions (HSU n=9, HSP n=4); audio recording & transcription; thematic analysis (extraction of statements re self management);	development of text content from identified credible resources using plain language writing style guide (expert team); preliminary website interface design; selection of communication channels eg text, video etc based on feedback from expert team & literature (HSP); development of interview guide; individual semi structured interviews (HSU n=5) & group semi structured interviews (HSU n=12) to help determine content & interface & provide feedback on preliminary content areas & website design;audio recording; [note that fig.1 shows these processes happening in tandem but it is unclear how early HSUs were involved in the process.]	prototype; final individual questionnaire for feedback on close to final draft website (HSU n=10); final prototype submitted to validation panel; validation panel (experts n- not defined); content finalised;	Not reported	Randomized Controlled Trial (RCT) called RAISIN (Randomized Trial of an Asthma Internet Self-management Intervention); ISRCTN 78556552
O'Brien 2016 [51]	LEAP (Living, Eating, Activity, and Planning in retirement) - web based lifestyle intervention in retirement	iterative co-design; behavior change techniques; Health Action Process Approach;	male & female adults aged 55+ as potential intervention users; different stages of retirement transition; diverse socioeconomic backgrounds;	health & social care professionals whose role related to improving health & well-being of people in retirement;	"Project Team: not defined;	Ethics approval; 3 x systematic reviews - literature search; expert evidence summary of literature reviews, qualitative research findings & expert knowledge to create "evidence statements";	iterative review of suggested website features; [value specification combined with contextual inquiry - see previous]	creation of video segments for websites (HSU and HSP); review of all content by health literacy experts; Health Literacy Advisor software to evaluate language; prototype; development of usability testing guide; iterative usability testing	Not reported	Pilot Randomized Controlled Trial (RCT) to be reported elsewhere

Detail of data extracted from 30 studies scoring ≥ 90% on MMAT (additional information may have been reported in literature that was not reviewed)										
Study / References	Specific product	Frameworks, models, theories	Health service user (HSU) population	Health Service Provider (HSP) population	Teams or groups	CeHRes roadmap: Contextual inquiry	CeHRes roadmap:Value Specification	CeHRes roadmap: Design	CeHRes roadmap: Operationalization	CeHRes roadmap: Summative Evaluation
								including interviews, observation and think aloud until no further changes were suggested (HSU n=12); audio recording; qualitative & quantitative data analysis; refinements to website in response to usability testing;		
Peute 2015 [52]	website for childhood cancer survivors	limited subset of Website Development Model for the Healthcare Consumer (WDMHC); user-centred design; iterative development methodology	cancer survivors and parents; youth aged 20-25; parents aged 50-55	Pediatrician + pediatric oncologist		literature review; structured questionnaire (HSU n= 145); information needs analysis resulted in list of information requirements for website;	Codesign workshop 1 to brainstorm new intervention ideas, including processes of persona building, experience mapping, wild cards, storyboarding (n=42 including research team n=12, HSU n=22, HSP n = 8); detailed notes by facilitators; thematic analysis;	low fidelity protype; iterative review of prototype by expert panel; iterative development of protoypes; iterative think aloud studies/interviews of early prototype - draft pages in Powerpoint (HSU n=8); think aloud studies/interviews of later prototype - prototype web pages (HSU n=3); audio recording; thematic analysis using NVivo software; used LifeGuide open access software;	Not reported	Not reported
Revenas 2015 [53-55]	"'tRAppen" - Swedish app for self management of physical activity in rheumatoid arthritis	participatory action research; experience based design (EBD); user centred design; participatory design; codesign	individuals diagnosed with rheumatoid arthritis for at least 1 year; Swedish communication skills; recruited from hospitals & members of Swedish Rhematism Association; age range 31-73	clinical pyshiotherapist;	Development Team: web designers & programmers"	Ethics approval; development of semi structured interview guide; testing of interview guide (physiotherapy students n=4); refinement of interview guide; 6 x focus group semi structured interviews based on Nominal Group Technique, including questionnaire re characteristics, self report form re readiness to engage in physical activity and group discussion (HSU n=26);	simple mock up website; focus group of stakeholders re expectations and feedback on mock up, with structured agenda & brainstorming (n=8 including HSU (n=4);, HSP (n= 2), IT experts (n=2); minutes of meeting;	Codesign workshop 2 to develop core intervention concepts using a creative facilitator to develop hand drawn prototypes including processes of persona building, mapping typical week , prototyping (n=20 including research team (n=6) and HSU (n=14); facilitator detailed notes; thematic analysis; hand drawn prototypes; design brief and specification document to inform intervention build (research team); Codesign	Not reported	Not reported

Detail of data extracted from 30 studies scoring ≥ 90% on MMAT (additional information may have been reported in literature that was not reviewed)										
Study / References	Specific product	Frameworks, models, theories	Health service user (HSU) population	Health Service Provider (HSP) population	Teams or groups	CeHRes roadmap: Contextual inquiry	CeHRes roadmap:Value Specification	CeHRes roadmap: Design	CeHRes roadmap: Operationalization	CeHRes roadmap: Summative Evaluation
								workshop 3 to de-risk the prototype, test & identify modifications using cognitive walkthrough (n=37 including research team (n=8) and HSU (n=29); analysis of feedback and issues identified in workshop to create list of revisions; optimized functioning prototype; iterative testing, user feedback and intervention refinement process of revised intervention prototype (n=30 including research team, HSU and HSP);		
Sandlund 2015 [56]	mobile exercise app to prevent falls	Participatory and Appreciative Action and Reflection (PAAR); Form-IT including Soft Systems Thinking; Needfinding; Appreciative Inquiry; Meta-design including Designing Design, Designing Together & Designing the "in between";	community living older persons recruited by senior citizen associations; heterogenous group with different experiences of technology, falls etc; age range 70-77 (n=18)	See research group	"Project steering group heads of university counselling services, charity representative, psychiatrist;	Ethics approval; Individual interviews to identify participants (HSU n=38); 1st workshop including presentation & focus group discussion, intro & personal meaning of joy of movement & balance (HSU n~18)	prioritization task (HSU n=22); audio recording & thematic analysis of results from focus groups & prioritization task - inductive qualitative content analysis; [Process to this point reported in detail in 2014 paper - described as 1st stage]	development of working prototype including established website usability guidelines (web design company); heuristic evaluation (double experts in usability & cancer survivorship care n=3); think aloud analysis including SUS questionnaire (HSU n=8); audio & screen recording; thematic analysis with Morae software	Not reported	Not reported
Schnall 2014 [57]	mobile app for HIV prevention for high risk MSM	Information Systems Research Framework (ISR) including 3 cycles - Relevance, Rigor & Design (includes diagram); iterative design; user-	Men reporting having unprotected sex with HIV positive or unknown status male partners; self reported HIV negative or HIV status unknown; able to			5 x focus groups identifying desired functions & design of app (HSU n=33); thematic analysis; literature review (published systematic review re existing technology in HIV prevention);	2nd workshop including 2 focus group discussions re personal meaning & avoiding falls (HSU n~18); 3rd workshop including lecture & focus group discussion re how new tech can inspire you (HSU n~18); 4th workshop including discussion in	pilot workshop to test data collection process; 1st co-design workshop aims & intro, brainstorming (co-design group n=8 inc 3 HSU); 2nd co-design workshop create 1st prototype, brainstorming & narrowing, creation of 1st framework (co-design	Not reported	Not reported

Detail of data extracted from 30 studies scoring ≥ 90% on MMAT (additional information may have been reported in literature that was not reviewed)										
Study / References	Specific product	Frameworks, models, theories	Health service user (HSU) population	Health Service Provider (HSP) population	Teams or groups	CeHRes roadmap: Contextual inquiry	CeHRes roadmap:Value Specification	CeHRes roadmap: Design	CeHRes roadmap: Operationalization	CeHRes roadmap: Summative Evaluation
		centred design	read and write in English or Spanish; age range 18-57;				small gender divided groups re activity strategies (HSU n~18); 5th workshop including activity & discussions in small gender divided groups re wishes for app (HSU n~18);	group n=10 inc 5 HSU); focus group interview re co-design process (co-design group n=10 inc 5 HSU); 3rd co-design workshop to create 2nd prototype (co-design group n=8 inc 4 HSU); 4th co-design workshop - feedback on 2nd prototype (co-design group n=8 inc 4 HSU); 2nd focus group interview re co-design process (co-design group n=8 inc 4 HSU); data collection of workshops using online notice board (Trello), interactive boards, post it notes, video recordings & observation protocols; video based analysis; thematic analysis; [this 2nd stage reported in detail in 2015 papers]		
Skjoth 2015 [58]	http://graviditetsportalen.dk/ - interactive website to support pregnant women in making informed choices about Downs Syndrome screening	participatory design; International Patient Decision Aid Standards (IPDAS) Collaboration Guide;Centre for ehealth Research and Disease Management (CeHRes) roadmap	pregnant women (some previously participated in Down syndrome screening); recruited from midwife consultations; Danish; age range 21-39;	3 care providers - consultant, nurse and midwife from Maternal Fetal Medicine Clinic at Odense University Hospital. 5+ years experience in maternal fetal medicine.	Professional expert validation panel: 3 psychologists, 1 psychiatrist ;	stakeholders identified; Research Group recruited; literature review (published systematic review); 1st focus group using semi structured guide (HSP n=3); 2nd focus group using semi structured guide (HSU n= 8);	Design session 1 including focus group methodology to identify features for improving HIV prevention in MSM (HSU n-6); audio recording & notes taken; thematic analysis; agreement on app content & use cases (study team);	6th workshop working with personas (HSU n~18); 7th workshop including feedback, dsicussions, activity (HSU n~18); development of 1st prototype; 8th workshop: test & feedback re 1st prototype & exercises (HSU n~18); 2nd prototype developed; 9th workshop test & feedback of 2nd prototype, visit to research lab re brainstorming app feedback, questionnaire on motivation re falls prevention (HSU n=16); 3rd prototype developed; 10th workshop - testing	website designed to reflect clinical pathway at Odense University Hospital;	Not reported

Detail of data extracted from 30 studies scoring ≥ 90% on MMAT (additional information may have been reported in literature that was not reviewed)										
Study / References	Specific product	Frameworks, models, theories	Health service user (HSU) population	Health Service Provider (HSP) population	Teams or groups	CeHRes roadmap: Contextual inquiry	CeHRes roadmap:Value Specification	CeHRes roadmap: Design	CeHRes roadmap: Operationalization	CeHRes roadmap: Summative Evaluation
								3rd prototype (HSU n~18); observation of participants during all workshops; content analysis; questionnaire re motivating factors in fall prevention to larger sample of potential users (HSU n=42); iterative prototype evaluation through continuing workshops (HSU n=38); use of ACKTUS platform;		
Stinson 2014 [59]	iCanCope with Pain - mobile based self management program for youth with chronic pain	user centred design; iterative design	adolescents recruited from pediatric chronic pain clinic in Ontario; pain lasting over 3 months with poor response to conventional pain management therapies; age range 14-18; able to speak & read English	Interdisciplinary health care providers recruited from pediatric chronic pain clinics in Ontario; employed full time for at least 1 year in pain clinics;		Ethics approval; demographic questionnaire (HSU n=23); sociodemographic questionnaire (HSP n=7); data analysis;	field observations and individual interviews (HSU + HSP - numbers not reported); 3rd focus group using content and interview guide (HSU n=6); audio recording; thematic analysis;	Design session 2 re user interface design, sketching ideas (HSU n=6) ; audio recording; thematic analysis; low fidelity prototype based on results from design sessions; heuristic evaluations (informaticians/IT experts n=5); usability testing including Post Study System Usability Questionnaire (PSSUQ) (HSU n=10); data analysis using Morae software & SPSS v22; iterative prototype development (5 versions);	Not reported	Not reported in this article, but this app was produced and evaluated in a Randomized Controlled Trial (RCT) https://www.clinicaltrials.gov/ct2/show/NCT02601755
van Bruinessen 2014 [60, 61]	PatientTIME; 3 products: self directed online communication tool, a corresponding evaluation plan and an implementation plan. Designed to empower patients to communicate with health providers.	Intervention Mapping Framework (IM) (includes diagram); patient participatory methods; participatory design; user centred design (UCD); participation ladder; iterative design; context map-	patients over 18 years old diagnosed with malignant lymphoma including Hodgkin's lymphoma and non-Hodgkin's lymphoma; age range 39-81; (patients who usually took a companion to appointments		Student user group (university students / HSU's but described differently throughout eg 'student consultants')"	ethics approval checked but not required; recruitment of patient research partners involved throughout with an initiating & agenda setting role (HSU n=2); creation of multidisciplinary working group (health professionals + 1 patient research partner); questionnaire about diagnosis (HSU n=28);	development of focus group interview guides; 3 x focus groups (HSU n=23); 1 x focus group (HSP n=7); audio recording; thematic analysis;	prototype; prototype evaluation (HSU n=6; HSP n=2); website evaluation using IPDAS checklist for developing patient decision aids; product refinement focusing on usability	Implementation plan developed in connection with Hematon - conditions built to transfer evaluated intervention to publicly available tool;during design process main criteria for selecting strategies was ability to operationalize in online	Evaluation plan; development of pretest RCT questionnaires using think aloud (HSU n=2); RCT protocol developed & audited with external experts; Randomized Controlled Trial (RCT). Netherlands

Detail of data extracted from 30 studies scoring ≥ 90% on MMAT (additional information may have been reported in literature that was not reviewed)										
Study / References	Specific product	Frameworks, models, theories	Health service user (HSU) population	Health Service Provider (HSP) population	Teams or groups	CeHRes roadmap: Contextual inquiry	CeHRes roadmap:Value Specification	CeHRes roadmap: Design	CeHRes roadmap: Operationalization	CeHRes roadmap: Summative Evaluation
		ping framework; stepwise protocol; Stanford Guidelines for Web Credibility; modelling;	could invite them to their interviews)						environment that could be hosted by Hematon; implementation planning started from first stages of project planning; Patient research partners (HSU n=2) to be involvedi n transfer of the intervention;	National Trial Register ID NTR3779
Widman 2016 [62]	ProjectHeartforGirls.com, interactive web based program designed to improve sexual communication skills and reduce risk of HIV/STDs among adolescent girls	Reasoned Action Model; fuzzy-trace theory	ethnically diverse adolescent high school students; female; aged 16-19;		"Interdisciplinary expert team: not defined;	formative qualitative research including semi-structured interviews re current sexual communication issues & recommendations for content (HSU n=25); audio recording; thematic analysis using Dedoose software;	needs assessment including assignments - booklet with questions about communicating with clinicians (HSU n=23); 3 x focus groups & 10 x interviews (HSU n= 28+9 companions); audio recording; thematic analysis using MAXQDA software; [This needs assessment recorded in detail in 2013 article]	program architecture / prototype developed; individual semi structured interviews re architecture (HSU n=7); audio recording; thematic analysis;	Not reported	Randomized Controlled Trial (RCT); Clinical Trials registration NCT02579135 (HSU n=222) - results not yet reported
Winterling 2016 [63-66]	Fex-Can, Fertility and sexuality following cancer	participatory design; CeHRes Roadmap (referred to as holistic framework for developing eHealth technologies by van Gemert-Pijnen et al)	depending on stage of research included newly diagnosed cancer patients or cancer survivors and significant others; age range 16-41; cancers included Hodgkin lymphoma (n=5), tumours of central nervous system (n-2), breast cancer (n-2), testicular cancer	health professionals with experience in primary care, psychotherapy & counseling in cancer care and sexually transmitted infections; physicians and nurse practitioners in cancer and reproductive care and sexual therapists		ethics approval for formative research; formative research via 39 x written online focus group discussions with cancer survivors aged 16-24 (HSU n=133); inductive qualitative content analysis/ thematic analysis of focus groups using NVivo software; individual semi structured interviews with 21 newly diagnosed cancer patients (HSU n=21); qualitative content analysis / thematic analysis of interviews;	[initial contextual interviews included value specification]; review of other websites to inspire design; literature review;	iterative design process adapting to feedback from HSU & HSP; rough content reviewed (HSU + HSP); reviews by patient research partners (HSU n=2): prototype development; heuristic evaluation (experts n=4); think aloud testing (HSU n=4);	PLANNED feasibility study with PRP to participate in interpretation of results (process and results not reported)	"Randomized Controlled Trial (RCT) - International Standard Randomized Controlled Trial Number (ISRCTN): 36621459;

Detail of data extracted from 30 studies scoring ≥ 90% on MMAT (additional information may have been reported in literature that was not reviewed)										
Study / References	Specific product	Frameworks, models, theories	Health service user (HSU) population	Health Service Provider (HSP) population	Teams or groups	CeHRes roadmap: Contextual inquiry	CeHRes roadmap:Value Specification	CeHRes roadmap: Design	CeHRes roadmap: Operationalization	CeHRes roadmap: Summative Evaluation
			(n-2), cervical cancer (n-1) & Ewing sarcoma (n=1)							
Ennis 2014 [67, 69]	myhealthlocker. Electronic personal health record (ePHR) for people with severe & enduring mental health problems	participatory design	adult users of mental health services including psychosis and other diagnoses; age range 18-65;	clinicians from psychosis services and primary care services	Research team: not defined other than as authors of another paper;	Ethics approval; creation of Advisory Board (HSU + HSP); development of interview schedules & survey questions collaboratively with Service User Advisory Group (HSU + HSP?); interviews re needs & priorities (HSU n=?); focus groups re needs & priorities (HSU n=?); audio recording & transcription of focus groups; survey of computer literacy & access (HSU n=121); data analysis of survey responses;	recruitment of paid PRP for feedback throughout (HSU n=13); initial 1 day meeting with PRP & RG to discuss collaboration preferences (HSU n=13);	design of logo & initial mock ups by designers; creation of Teen Advisory Board for ongoing feedback (HSU n=5); iterative drafting & piloting of intervention content by 7x meetings with Teen Advisory Board (HSU n=5); recording of audio clips as examples of good communication by Teen Advisory Board (HSU n=5); iterative technical development by Research Group in collaboration with Communication for Health Applications and Interventions (CHAI) Core over 12 months (experts); usability testing using think aloud protocol (HSU n=6); resulting changes to final product;	creation of service user planning committee re dissemination (HSU n=4); weekly drop in sessions at local community venues with end user co-facilitators (HSU n-3); feasibility questionnaire WEMWBS PROM (HSU n=23);	http://www.isrctn.com/ISRCTN36621459. Results not yet reported."
Fleisher 2014 [68]	PRE-ACT (Preparatory Education About Clinical Trials)	Cognitive Social Health Information Processing Theory; Ottawa Decision Support Framework	cancer patients and patient advocates; age range 40-70;	none defined		survey re barriers to decision making (HSU n=156);	[combined with contextual inquiry processes - see previous];	4 x 1-day meetings with PRP & RG during design step including plenary, small group discussions & individual assignments, meeting minutes & research reflections after (HSU n=12); log book of meeting minutes; iterative development between meetings including creation of mock up and prototype;	not reported	
