# Supplementary material for: Participatory Methods to Engage Health Service Users in the Development of Electronic Health Resources: Systematic Review
Source: J Particip Med. 2019 Feb 22;11(1):e11474. doi: 10.2196/11474 (PMC7434099; doi:10.2196/11474)
Supplement: Multimedia Appendix 9 [file jopm_v11i1e11474_app9.pdf]

Study and references	Health area (MeSH[a])	Purpose (MeSH)
Ahtinen 2013 [26]	Health promotion	Exercise, Health Behavior, Healthy Lifestyle, Motivation
Antypas 2014 [27]	Cardiovascular Diseases	Cardiac Rehabilitation, Exercise, Health Behavior
Bengtsson 2014 [28,29]	Cardiovascular Diseases	Self-care
Buccieri 2015 [30]	Health Promotion	Homeless Persons, Health Services Accessibility
Clayman 2008 [31]	Breast Neoplasms	Patient Education as Topic
Cordova 2015 [32]	Virus Diseases, Sexually Transmitted Diseases, HIV, Substance-Related Disorders	Health Promotion, Power (Psychology), Primary Health Care
Dabbs 2009 [33]	Respiratory Tract Diseases, Lung Transplantation	"Health Records, Personal"
Das 2013 [34]	Nutritional and Metabolic Diseases	Food and Nutrition, Weight Reduction Programs, Follow-Up Studies
Davies 2015 [35,36]	Virus Diseases, Hepatitis B	Patient Education as Topic, Health Promotion
Fennell 2016 [39,44]	Neoplasms	Psychosocial Support Systems, Health Services Accessibility
Fonda 2010 [40,41]	Endocrine System Diseases, Diabetes Mellitus	Self care, Patient Education as Topic, "Health Records, Personal"
Goldenberg 2015 [42,43]	Virus Diseases, Sexually Transmitted Diseases, HIV	Patient Education as Topic
Heckman 2015 [45]	Skin Neoplasms	Patient Education as Topic, Health Behaviour
Kelders 2013 [46]	Mental Disorders, Depressive Disorder	Behaviour Therapy, "Acceptance and Commitment Therapy", Patient Education as Topic
Lubberding 2016 [37,38,47]	Neoplasms	Survivors, "Quality of Life", Self care, Healthy Lifestyle, Psychosocial Support Systems
Meyer 2007 [48]	Mental Disorders, Depressive Disorder	Power (Psychology), Self care, "Patient Acceptance of Health Care"
Miller 2015 [49]	Prostatic Neoplasms	Survivors, Self care, "Adaptation, Psychological"
Morrison 2015 [50]	Respiratory Tract Diseases, Asthma	Self care
O'Brien 2016 [51]	Geriatrics	Retirement, Patient Education as Topic
Peute 2015 [52]	Neoplasms	Survivors, Patient Education as Topic
Revenas 2015 [53-55]	Musculoskeletal Diseases, Rheumatoid Diseases, "Arthritis, Rheumatoid"	Exercise, Self care
Sandlund 2015 [56]	Geriatrics	Accidental Falls, Exercise, Motivation
Schnall 2014 [57]	Virus Diseases, Sexually Transmitted Diseases, HIV	Patient Education as Topic, Health Behavior
Skjoth 2015 [58]	Women's Health	Patient Education as Topic, Decision Making, Decision Support Techniques, Down Syndrome, Prenatal Diagnosis
Stinson 2014 [59]	Nervous System Diseases, Pain; Chronic Pain	Self care, Psychosocial Support Systems, "Adaptation, Psychological", Patient Education as Topic
Van Bruinessen 2014 [60,61]	Neoplasms, Lymphoma	Communication, Physician-Patient Relations, Patient Education as Topic
Widman 2016 [62]	Virus Diseases, Sexually Transmitted Diseases, HIV	Sex Education, Communication, Sexual Behaviour, Health Behaviour
Winterling 2016 [63-66]	Neoplasms	Sex Education, Reproductive Health, Self care, Patient Education as Topic
Ennis 2014 [67,69]	Mental Disorders	"Health Records, Personal"
Fleisher 2014 [68]	Neoplasms	Patient Education as Topic, Decision Making, Decision Support Techniques

[a]MeSH: Medical Subject Headings used in MEDLINE
