# Supplementary material for: Participatory Methods to Engage Health Service Users in the Development of Electronic Health Resources: Systematic Review
Source: J Particip Med. 2019 Feb 22;11(1):e11474. doi: 10.2196/11474 (PMC7434099; doi:10.2196/11474)
Supplement: Multimedia Appendix 10 [file jopm_v11i1e11474_app10.pdf]

Model or theory base (extracted from top 30 studies and searched across full text of 90 studies in Endnote)	Total studies scoring ≥ 90% on MMAT n=30 (%)	Total MMAT rated studies n=90 (%)	References (studies may include more than one reference)
Social Cognitive Theory [145]	4 (13%)	11 (12%)	[26, 27, 39, 55, 70, 104, 117, 126, 134, 137, 142, 143]
Theory of Planned Behaviour [146]	3 (10%)	7 (8%)	[27, 39, 62, 99, 104, 113, 115]
Transtheoretical Model (Prochaska Stages of Change) [147]	3 (10%)	6 (7%)	[26, 27, 39, 101, 105, 140]
Persuasive Technology Theory / Behaviour Model for Persuasive Design [148]	3 (10%)	6 (7%)	[27, 46, 61, 89, 96, 106]
Health Behaviour Theory [157]	3 (10%)	4 (4%)	[27, 55, 62, 81]
Cognitive-Social Health Information Processing (CSHIP) Model	2 (7%)	3 (3%)	[49, 68, 125]
Business modelling	2 (7%)	2 (2%)	[46, 58]
Health Action Process Approach (HAPA)	2 (7%)	2 (2%)	[27, 51]
Health Belief Model (HBM)	1 (3%)	3 (3%)	[39, 84, 94]
Common Sense Model	1 (3%)	2 (2%)	[28, 138]
Reasoned Action Model	1 (3%)	2 (2%)	[62, 99]
Appreciative Inquiry (AI)	1 (3%)	1 (1%)	[56]
Behavioural Science Theory	1 (3%)	1 (1%)	[49]
Critical Social Theory of Youth Empowerment	1 (3%)	1 (1%)	[30]
Empowerment Theory	1 (3%)	1 (1%)	[32]
Fuzzy-trace Theory	1 (3%)	1 (1%)	[62]
Information-Motivation Behavioural Skills Model	1 (3%)	1 (1%)	[57]
Integrative Model of Behaviour Prediction	1 (3%)	1 (1%)	[45]
Normalization Process Theory (NPT)	1 (3%)	1 (1%)	[50]
Ottawa Decision Support Framework	1 (3%)	1 (1%)	[68]
Paasche-Orlow & Wolf's Model (causal pathways)	1 (3%)	1 (1%)	[36]
Regulatory Focus Theory (RFT)	1 (3%)	1 (1%)	[27]
Social Constructionist Philosophy	1 (3%)	1 (1%)	[48]
