# Supplementary material for: Participatory Methods to Engage Health Service Users in the Development of Electronic Health Resources: Systematic Review
Source: J Particip Med. 2019 Feb 22;11(1):e11474. doi: 10.2196/11474 (PMC7434099; doi:10.2196/11474)
Supplement: Multimedia Appendix 11 [file jopm_v11i1e11474_app11.pdf]

**Participatory framework or approach (extracted from top 30 studies and searched across the full text of 90 studies in Endnote)**	**Total studies scoring ≥ 90% on MMAT n=30 (%)**	**Total MMAT rated studies n=90 (%)**	**References (studies may include more than one reference)**
**Named Framework**			
User Centred Design (UCD) [149]	5 (17%)	10 (11%)	[33, 34, 41, 52, 61, 90, 91, 106, 109, 133]
Participatory Action Research (PAR) Framework [154]	4 (13%)	5 (6%)	[30, 35, 36, 39, 53, 55, 100]
Centre for ehealth Research and Disease Management (CeHRes) Roadmap [25]	3 (10%)	6 (7%)	[17, 46, 58, 63, 95, 133]
Medical Research Council (MRC) Guide to Developing and Evaluating Complex Interventions [150-152]	2 (7%)	6 (7%)	[50, 51, 76, 118, 119, 125]
International Patient Decision Aid Standards (IPDAS) Collaboration [153]	2 (7%)	5 (6%)	[58, 68, 81, 121, 141]
Human Centred Design (HCD)	2 (7%)	4 (4%)	[6, 34, 46, 56]
Action Research Framework	2 (7%)	2 (2%)	[39, 48]
Agile Software Development	1 (3%)	4 (4%)	[32, 82, 100, 140]
Intervention Mapping Framework (IM)	1 (3%)	4 (4%)	[61, 113, 130, 134]
Community-based Participatory Research (CBPR)	1 (3%)	3 (3%)	[32, 92, 101]
Collaborative Analysis of Requirements and Design (CARD)	1 (3%)	1 (1%)	[34]
Constructive Design Research	1 (3%)	1 (1%)	[26]
Context Mapping Framework	1 (3%)	1 (1%)	[60]
Ecodevelopmental Framework	1 (3%)	1 (1%)	[32]
Experience Based Design (EBD)	1 (3%)	1 (1%)	[53]
Form-IT	1 (3%)	1 (1%)	[56]
Information Systems Research (ISR) Framework	1 (3%)	1 (1%)	[57]
Participatory and Appreciative Action and Reflection (PAAR)	1 (3%)	1 (1%)	[56]
Persuasive System Design (PSD) Model	1 (3%)	1 (1%)	[26]
Soft Systems Thinking	1 (3%)	1 (1%)	[56]
Spiral Technology Action Research (STAR) Model	1 (3%)	1 (1%)	[30]
Stanford Guidelines for Web Credibility	1 (3%)	1 (1%)	[61]
Website Development Model for the Healthcare Consumer (WDMHC)	1 (3%)	1 (1%)	[52]
Youth Centred Participatory Action Study	1 (3%)	1 (1%)	[30]
**Broad approaches described (indicating language used)**			
“participatory design” or “participatory development”	8 (27%)	17 (19%)	[26, 34, 37, 38, 47, 53-58, 63, 81, 86, 91, 102, 110, 120, 123, 128, 138]
“iterative design” or “iterative development”	7 (23%)	12 (13%)	[26, 34, 52, 57, 61, 78, 91, 106, 109, 110, 116, 128, 137]
