# Supplementary material for: Participatory Methods to Engage Health Service Users in the Development of Electronic Health Resources: Systematic Review
Source: J Particip Med. 2019 Feb 22;11(1):e11474. doi: 10.2196/11474 (PMC7434099; doi:10.2196/11474)
Supplement: Multimedia Appendix 12 [file jopm_v11i1e11474_app12.pdf]

**Interventions (extracted from top 30 studies and searched across the full text of 90 studies in Endnote)**	**Total studies scoring ≥ 90% on MMAT n=30 (%)**	**Total MMAT rated studies n=90 (%)**	**References (studies may include more than one reference)**
Cognitive behaviour therapy (CBT)	5 (17%)	14 (16%)	[39, 48, 50, 59, 67, 69, 80, 89, 95, 102, 109, 111, 118, 119, 122, 125, 134, 137]
Behaviour change techniques (BCT's)	4 (13%)	10 (11%)	[39, 50, 51, 54, 55, 70, 81, 115, 137, 140, 142, 143]
Mindfulness	4 (13%)	8 (9%)	[46, 59, 63, 67, 95, 109, 125, 134]
Acceptance and commitment therapy (ACT)	2 (7%)	3 (3%)	[39, 46, 95]
National Comprehensive Cancer Network Distress Management Guidelines	1 (3%)	1 (1%)	[39]
Prevention principles of the National Institute on Drug Abuse (NIDA)	1 (3%)	1 (1%)	[32]
