# Supplementary material for: Participatory Methods to Engage Health Service Users in the Development of Electronic Health Resources: Systematic Review
Source: J Particip Med. 2019 Feb 22;11(1):e11474. doi: 10.2196/11474 (PMC7434099; doi:10.2196/11474)
Supplement: Multimedia Appendix 13 [file jopm_v11i1e11474_app13.pdf]

Study [ref]	Themes / Findings (HSU perspective)	Author/Researcher recommendations
Ahtinen 2013 [26]	1. **Be my advisor**: personal trainer that supports and motivates wellness activities<br>2. **Acknowledge my efforts**: show progress and provide encouraging feedback<br>3. **Grow with me:** evolve and adapt programs, activities and challenges<br>4. **Keep me engaged:** introduce new features to retain interest	1. Design of research can be successfully applied to motivate and support wellness activities<br>2. The four design principles are relevant for motivating physical activity but cultural consideration is required<br>3. Individual advising, encouraging and evolving as structural components of the program (received well by both cultures)<br>4. Mobile phones as an important platform for applications in health and wellness. They support: real time data collection; monitoring; analysis of health information; personalisation in wellness application
Antypas 2014 [27]	1. **Social**: companionship, communication with others<br>2. **Motivation:** ideas and methods influencing the participants' behaviour<br>3. **Integration to everyday life**: helping participants to integrate a desirable behaviour into everyday life<br>4. **Information**: access to information after discharge<br>5. **Planning**: plan physical activity in advance in order to complete<br>6. **Monitoring and feedback:** keeping and presenting a record of physical activity in meaningful way<br>7. **Concerns and potential problems**: lack of time, training facilities, literacy	1. Combination of Health Behaviour Theory and user input is a promising design approach<br>2. Communication design of a website is an essential component of the intervention<br>3. Both prioritisation and motivation are needed to make sure each participant will be ready to overcome any difficulty<br>4. Self-efficacy is the most important variable in Social Cognitive Theory. Social variables play several important roles in the stages of behavioural change
Bengtsson 2014 [28, 29]	1. **Framing outcomes in order to self-manage treatment:** a) communicating and understanding high blood pressure and its treatment; b) Perceptions of high blood pressure and its treatment eg trust, good relationship with caregivers and well-being<br>2. **Measuring outcomes in order to self-manage hypertension:** a) measurement to be followed; b) making use of measuring outcomes e.g. Blood pressure, dizziness, stress, headache and tiredness	1. HSP highlighted accessibility, clear and consistent counseling, complication prevention and educational efforts<br>2. Importance of follow-up to understand interplay between blood pressure and daily life experiences of patients with hypertension<br>3. Focus group to be representative of greater population<br>4. Mobile phone self-report system is reliable to capture information relevant to hypertension self-management
Buccieri 2015 [30]	1. **Welcoming/ safe environment**: meetings took place in the basement of a youth shelter<br>2. **Combine technology / access and information to empower**: app improve access to information and services<br>3. **Treat with respect / taken seriously:** potential employment opportunities<br>4. **Combatting stigmatisation:** homeless youth are capable individuals and not treated as less of a person<br>5. **Engage in critical analysis and sense of pride**: sense of pride and ownership during the development of the app	1. Restriction on money, time and technological resources meant some ideas not practical<br>2. Making the program widely accessible by delivering it on a variety of platforms e.g. mobile phone and website<br>3. Technology improved communication between homeless youth and support workers and created a solution focused environment<br>4. Research environment created a unique experience for young people<br>5. Further research needed to understand mobile technology uptake for homeless youth
Clayman 2008 [31]	1. **Easy to understand, relevant information:** information presented under 6 key questions	1. Combining patient, provider and clinical perspective to create an education program that

Study [ref]	Themes / Findings (HSU perspective)	Author/Researcher recommendations
	2. **Improve coordination of care, patient- centered information design**: information on website embraced by patients and endorsed by physicians<br>3. **Limit the overwhelming resources/types of information**: create basic information with a clear and consistent style with appropriate links<br>4. **Interactive dynamic flowchart**: flowchart that allows women to see the treatment options relevant for their diagnosis	provides clear and accurate information<br>2. Form: website; immediate access to information and provides level of continuity<br>3. Process: centrality of patient perspective<br>4. Content: addressing patients' information and support needs
Cordova 2015 [32]	1. **A mHealth app for adolescents in primary care:** time, place platform and length of program to maintain engagement<br>2. **Risk assessment to improve clinician-adolescent HIV/STI, drug use communication:** facilitate difficult conversations<br>3. **Culturally specific HIV/STI, drug use content:** aim to reduce fear and harm, educate<br>4. **Interactive aspects of the app to engage youth with visual/audio:** games and quizzes to reinforce information<br>5. **Appearance of the app:** aesthetically appealing, avoiding text heavy sections	1. Community-engaged approach to gather data in primary care<br>2. Need for secure platform for health information<br>3. Apps as an effective means of preventing and reducing HIV/STI risk behaviours<br>4. Include parents in prevention programs and to improve parent-adolescent HIV/STI communication<br>5. Collect clinician perspective as well as patient perspective<br>6. MHealth modalities have the potential to facilitate clinician-adolescent HIV/STI and drug use communication
Dabbs 2009 [33]	**1.** **Use of PATH:** daily use over 2-week period<br>**2.** **Checklist, logs and graphs:** daily use<br>**3.** **Normal thresholds set for clinical parameters:** feedback messages generated when values fell outside acceptable range<br>**4.** **Accessed message history/notes:** at least once<br>***5.*** **Technical support available:** to support upload of data	**1.** Pilot study determined Pocket PATH superior to conventional methods for self-monitoring after lung transplantation<br>**2.** Further research required to examine utility and functionality of Pocket PATH<br>**3.** Principles of UCD increases the likelihood of success promoting self-monitoring
Das 2013 [34]	1. **Gathered domain knowledge:** understand treatment and workplace environment<br>2. **Patient trajectory and medical treatment options:** understanding the appropriate stage to implement initiative<br>3. **Patient education and self-management:** promoted by future e-health solution<br>4. **Scenario building/patient personas:** provided valuable information on current practice and future solutions<br>5. **Reflection on user requirements:** insight into personal stories and daily life<br>6. **Redesign and modification**: medical content and redesign provided by obesity clinic and research team<br>*7.* **Usability testing and reflection:** helpful feedback for final improvement	1. Identify the stakeholders and context of use<br>2. Include the relevant stakeholders and context of use throughout the design process<br>3. Conduct homogenous workshops and design activities support participants engagement<br>4. Particular considerations and facilitation is required when including vulnerable user groups in UCD<br>5. Conduct usability evaluations as part of the system development process
Davies 2015 [35, 36]	1. **Local language:** accessible with either English or Yolngu Matha<br>2. **Visual and interactive:** animation and games included	1. Community partnerships and PAR methodologies can break down barriers to communication<br>2. Value can be gained from repeated reviews of and conversations during development process

Study [ref]	Themes / Findings (HSU perspective)	Author/Researcher recommendations
	3. **Tell the full /true story:** back translation conducted with focus on conceptual and cultural equivalence<br>4. **Accessible:** free download from Apple Store<br>*5.* **Cultural respect:** separate women's section requested	3. Obtaining balance between cultural appropriateness and community wishes versus inclusivity of potentially underrepresented groups
Fennell 2016 [39, 44]	1. **Website acceptability:** reported relevant to their needs, necessary and helpful<br>2. **Perceived Impact:** reported feeling less distressed and isolated<br>3. **Accessible:** reported ease of use<br>4. **Informative:** provided new knowledge	1. Use of technology to deliver health information in rural populations has great potential (wide reach, low cost)<br>2. Base program upon both literature and lived experience<br>3. Perceived expertise of authors can predict 'active trust' in online interventions and thus behavioural impact<br>4. Anonymity of online resource can add to appeal<br>5. Need for cultural and behavioural appropriateness in context of target audience<br>6. Improve health literacy for HSU and HSP<br>7. Employ means of evaluating success of program once administered<br>8. Large sample size required to represent greater population
Fonda 2010 [40, 41]	Prototype description<br>1. **Overview:** data repository provides analysis and recommendations based on clinical guidelines<br>2. **Original Implementation of Gadgets:** iGoogle portal displays the Gadgets based on diabetes self-management<br>3. **Nutrition and Physical Activity:** logging of meals nutritional information, caloric and physical targets,<br>4. **Glucose:** displays graph of glucose monitoring<br>5. **Medications:** generates reminder<br>6. **"What if Analysis":** feedback on how activity and diet impact on glucose levels<br>7. **Mood:** plotting mood against glucose levels<br>8. **Diabetes "Tip of the Day":** users preference in topic area and medium of delivery providing recommendations and reminders<br>9. **Original Strategy for Retrieving Self-management Data:** data retrieved from user entry and external self-management data	1. PHA interaction must be dynamic in nature, promote discovery and evolve with changes in the user's lifestyle and environment<br>2. Feedback and recommendation tools should be fresh and contextually and temporally relevant<br>3. Prototype to be able to: discover causes in the world of the user; infer causes of novel input or recognise behavioural pattern shifts; make predictions; direct behaviour
Goldenberg 2015 [42, 43]	1. Phase 1: education; interactive engagement; social networking; privacy and confidentiality<br>2. Phase 2: customisation/personalisation with reminders and sex diaryising and authority<br>3. Design/functionality<br>4. Perceived impact<br>5. Relatable vs professional<br>6. Sharing data<br>7. Stigma<br>8. Target population	1. When building a mobile HIV prevention app, interactive and community-centered process to collect data is vital<br>2. App-based intervention addresses a large and diverse risk group<br>3. Mhealth interventions should be guided by existing theories of behaviour change<br>4. Personalisation along with interactive functionality has been found to allow users to take ownership of app (correlative with Social Cognitive Theory of

Study [ref]	Themes / Findings (HSU perspective)	Author/Researcher recommendations
	9. Testing barriers/facilitators<br>10. Willingness and motivations: easy to use and understand; app promotion and advertising suggested to assist motivation	self-regulation)<br>5. Building an app takes time and resources
Heckman 2015 [45]	1. Interactive with avatar development<br>2. Tailored and understandable<br>3. Utilize multiple media formats<br>4. Maximize participant engagement	1. Gather input from multi-disciplinary experts and the intended population during the entire development process<br>2. Use mixed methods to provide the most complete and useful information<br>3. Plan, manage and revise plans, procedures, the website, and potentially the budget and timeline as the project progresses<br>4. The project was guided by intervention development and assessment guidelines for behavioural therapy, web-based intervention and health communication programs including heath literacy best-practice
Kelders 2013 [46]	1. **Professional support and feedback:** provide feedback on progress<br>2. **System:** application needs to be user friendly<br>3. **Content:** be effective and add value<br>4. **Service:** receiving care through technology<br>5. **Peer support:** a need for contact with others using the application	1. Application to encapsulate; holistic view, incorporating system/content/service/perspectives and values of different stakeholders<br>2. Overlap of user-based and expert-based interventions an important methodological advantage<br>3. Iterative stage provided insight in the goals and technology that was being developed<br>4. Involve stakeholders at all stages<br>5. Wider demographic for the focus group with greater number of participants<br>6. Long term study to assess effectiveness of program
Lubberding 2016 [37, 38, 47]	1. **Attention paid to symptoms post treatment:** duration, variation and specification of symptoms<br>2. **Current referral to supportive care:** supportive care services referred; physical therapist, psychology, dietician, dental hygienist<br>3. **Monitoring quality of care:** feedback about results<br>4. **Personalised advice:** reliable information tailored to specific symptoms/patient<br>5. **Supportive care services:** access and availability to a variety of supportive care services	1. Adoption rate of ehealth can be predicted by the way an ehealth application is rated in terms of usefulness, ease of use and self-efficacy<br>2. Length of application and information overload can increase disconnect<br>3. Mixed methods provided in-depth insight into feasibility in clinical practice<br>4. Access was limited to 2 weeks and requires extension of access timeframe
Meyer 2007 [48]	1. Feedback from HSU were reflected in the website updates<br>2. Satisfied that website is professional, welcoming and positive impact for people<br>3. Site usage doubled in second year (100,000 visits) with consistent ratings of very or quite useful by HSU	Using SCP produced a far richer more accessible resource by:<br>1. Externalising language<br>2. Finding strategies, discussing illness in terms of a habit<br>3. Debating benefits and drawbacks of illness model of depression<br>4. Critically evaluating social and cultural influences to provide potential for options<br>5. Encouraging an activist or resistance position<br>6. Including stories of lived experienced framed within

Study [ref]	Themes / Findings (HSU perspective)	Author/Researcher recommendations
		strategies
Miller 2015 [49]	1. **Physical function:** practical advice, health concerns, treatment side effect concerns and health behaviours<br>2. **Emotional wellbeing:** body image, psychosocial issues<br>3. **Interpersonal concerns:** disruption of relationships with spouse/partner<br>4. **Practical barriers:** discriminatory workplace practices	1. Self-management focus of PROGRESS offers scalable, psychosocial intervention in addition to clinical care<br>2. Tailor website to cater to a wider demographic e.g. more language options<br>3. Address limitation of the information not being systematically collected concerning receipt of hormone therapy<br>4. Integrate PROGRESS support system with clinical health record to evaluate function<br>5. Desire for concrete strategies for managing treatment side effects
Morrison 2015 [50]	1. Information relevant and understandable<br>2. Layout and navigation of pages and sections needed refinement<br>3. HSP recommendation to support and promote website	1. Multifaceted approach results in online intervention relevant to HSU needs and ease of use<br>2. Iterative processes allowed website modification of website based on thinking aloud feedback<br>3. Focus group to be more representative of greater population<br>4. Qualitative evaluation involving participant interviews and using NPT to observe application in real world<br>5. Use of LifeGuard software allowed for streamlined and iterative process
O'Brien 2016 [51]	1. **Self-reflection:** in relation to financial, time, social, health and community resources<br>2. **Personalization:** a flexible intervention with individual feedback and tailored mentor support<br>*3.* **Social relationships**: reduce risks of social isolation, promote a sense of social support and share experiences in an engaging way	1. Co-design techniques involving stakeholders as an effective methodology<br>2. Further work required to apply web-based lifestyle intervention to other areas of health to refine and build evidence of usability and acceptability<br>3. Test feasibility and acceptability of LEAP in greater population
Peute 2015 [52]	1. Additional information needed: diagnosis, treatments, late effects, symptoms, therapies, rights as a patient and self-care<br>2. Discussion boards should be separate from the websites visited by HSP for privacy<br>3. Communication should be in a conservative manner to focus on reassurance instead of increasing anxiety<br>4. Navigational issues identified	1. Study findings supported the hypothesis that heuristic evaluation is sufficient for the development of WDMHC<br>2. Choice of methodologies used for specific phases of development process should be adapted to characteristics and needs of individual projects<br>3. Recommends live meeting with stakeholders early in development process
Revenas 2015 [53-55]	1. **Content:** up-to-date and evidence-based information and instruction, self-regulation tools and social interaction<br>2. **Customized options:** personalized set-up<br>3. **User interface:** attractive design and content<br>4. **Access and implementation:** access to the internet service and prioritized core features to include in a future internet service	1. The HSU, roles and responsibilities, aims, scope, to be clarified early in the project<br>2. Video recordings, protocols and naturalistic observations of co-design workshops provided triangulation of data and repeated viewing of videos<br>3. Consideration required to retain confidentiality due to video recordings<br>4. Task conflict was observed within the co-design and relied on participants ability to merge their perspectives

Study [ref]	Themes / Findings (HSU perspective)	Author/Researcher recommendations
Sandlund 2015 [56]	1. **Balance and joy of movement:** appreciating what this may look like for each individual<br>2. **Exercise preferences:** experiment with exercises participants liked and disliked<br>3. **Motivation:** factors that start and continue exercise amongst participants<br>*4.* **Design and development:** how the exercises are presented and to the user interface	1. Important learning generated through the Form-IT and PAAR methodology for HSU and HSP<br>2. App produced is easily adaptable to future needs and knowledge evolvement<br>3. Need for inclusion of motivating personalised feedback progressed over time<br>4. Transform exercises developed for the application into an interactive video game<br>5. Further evaluation in randomised controlled trials
Schnall 2014 [57]	1. **Information management:** log of past partners<br>2. **Staying healthy:** HIV information and prevention, diet/fitness<br>3. **HIV testing:** testing site information, testing log<br>4. **Chat / Communication:** medical providers, social/peers<br>*5.* **Resources:** support group locations, preventative measures, news	1. ISR framework supports: iterative process, needs assessment, functional requirement identification and user interface design and rapid prototyping<br>2. Future research required to understand effectiveness of the app and collection of more extensive usage and outcome data<br>3. Multistage approach is time consuming and costly<br>4. Iterative nature of framework allows for HSU and HSP feedback
Skjoth 2015 [58]	1. Difficulties in making an informed choice<br>2. Need for knowledge/information and where to find it<br>3. No understanding of cut-off values<br>4. Assessment of available information	1. Using IPDAS standards for developing patient decision aids helped ensure quality of content<br>2. Combining IPDAS with CeHRes roadmap provided a focused and structured developmental process was<br>3. Focus group interviews helped provide knowledge and understanding; effective method in the process of developing intervention<br>4. Important to maintain and update website for sustainability<br>5. Important to consider website as a supplement to face-face consultations<br>6. Cater to a greater demographic e.g. other languages
Stinson 2014 [59]	1. **Pain impact**: physical impact, role functioning, social/emotional impact and future<br>2. **Barriers to care**: healthcare system, patient-specific and societal<br>3. **Pain management strategies**: support system, pharmacological, physical, psychological<br>4. **Transition to adult care**: disconnect between pediatric and adult practices, skills needed to transition, parental role, fear and anxiety	1. Rigor of study enhanced by using analyst triangulation<br>2. Exploring perceptions of youth with chronic pain was crucial in laying foundation for iCanCope with Pain<br>3. Programs are a complement to professionally delivered health care and do not replace professional services<br>4. The web-based technology provide a more private, socially acceptable and normalising method of obtaining treatment in a less-stigmatising manner<br>5. Theoretical rationale supports self-monitoring, goal-setting, engaging with social support and improving health knowledge
Van Bruinessen 2014 [60, 61]	**1.** **Credibility:** information about collaborating parties, help function and privacy issues is missing/unclear<br>**2.** **Functionality:** Print function and use of agenda not clear<br>**3.** **Navigation:** not always clear where you are in	1. IM framework helped decision when to involve patients<br>2. UCD helped decision how to involve patients<br>3. Early involvement supported participation of patients<br>4. Flexibility required to incorporate patient

Study [ref]	Themes / Findings (HSU perspective)	Author/Researcher recommendations
	website and log-in button confusing<br>**4. Information:** some text too formal and inconsistency with terms/jargon<br>**5. Layout:** illustrative pictures too positive/happy, video clips unclear	viewpoints and experiences and include methodologies that allow dynamics of design such as Participatory Learning and Action Research or Design Inclusive Research
	1. **Design of the site:** color palate and illustration style<br>2. **Clear wording:** elucidation of key terms<br>**3. Addressing traditional myths**<br>4. **Emphasis on relationship skills:** including tips for identifying healthy and unhealthy relationships	Strength of project based on:<br>1. Psychological and health behaviour change theories<br>2. Focus on communication as central organising construct<br>3. Targeted audience with teen advisory board<br>4. Iterative approach to development<br>5. Usability testing with revision at several points in the process
Winterling 2016 [63-66]	1. **Information:** facts about treatments, side-effects and symptoms<br>2. **Content:** related to bodily changes or body image targeted at young cancer patients<br>3. **Exercises:** types of exercises, time dedicated to them, usefulness and reliability considered<br>4. **Language:** inclusive, easily comprehensible language matching a broad group of end users<br>5. **Awareness:** of participants' cancer experiences; include other patients' stories<br>6. **Feedback**: e-mail reminders, timeline, interactive quizzes<br>7. **Communication:** discussion forum and counseling<br>*8.* **Appropriateness:** addressing a diversity of sexualities, ethnicities, relationships and ways of building a family	1. Long-term collaboration over 5 years with Patient Research Partners (PRP) was highly successful<br>2. The system quality was improved by PRP feedback on design, technical malfunctions, and navigation on the website<br>3. PRP input made the content of Fex-Can meaningful, relevant and understandable.<br>4. Strategies to enhance PRP engagement included:<br>a) One person central contact<br>b) Established expectation of PRP roles<br>c) PCP seen as experts on patient perspective<br>d) Compensation for PRP time and expenses<br>e) Researcher and PRP reached common agreement
Ennis 2014 [67, 69]	1. **Information:** information about care, medication and side-effects<br>2. **Self-management:** monitoring own health and wellbeing<br>3. **Confidentiality:** sensitivity and security of information<br>4. **Social:** connected and interactive; allowing contact with clinician and others through social networking<br>*5.* **Presentation:** simple, minimal interface	Four key considerations for future developers of ehealth are:<br>1. Appeal – currently developing secure conference facilities for appointment scheduling and viewing test results<br>2. Construction - considering shared health information that is moderated in a secure way<br>3. Ease of use – clarity of navigation is critical with an intuitive system<br>4. Implementation – shift in attitudes is required for implementation in clinical practice and allay resistance and improve utility
Fleisher 2014 [68]	1. **Prepared me to ask questions:** preparing patients with questions prior to clinical trials<br>2. **Formatting:** easy to read, simple, not overwhelming<br>3. **Videos:** inclusion of videos to be short and informative<br>4. **Language:** easy to understand, caters to all education levels<br>5. **Informative:** informational without being boring, empowers decision making, helpful	1. HSU's perspectives need to be from early stage from conceptualization, development of scripts and selection of actors<br>2. Collaborative process is labour and time intensive process<br>3. Importance of creating interesting, multi-media approaches to engage patients in a serious and difficult topic<br>4. Multi-disciplinary team valuable to develop a theory-based sophisticated tool to assist decision

Study [ref]	Themes / Findings (HSU perspective)	Author/Researcher recommendations
	6. **Convenient:** being able to access at home<br>7. **Reassurance and hope:** comforting, inspire a sense of curiosity, empowerment<br>8. **Number of questions:** shorter and less questions<br>9. **Technicalities:** ensuring technical issues i.e. videos not loading, are minimised<br>10. **Tailoring**: tailor program to patients' background	making
